# Induced Microglial-like Cells Derived from Familial and Sporadic Alzheimer’s Disease Peripheral Blood Monocytes Show Abnormal Phagocytosis and Inflammatory Response to PSEN1 E280A Cholinergic-like Neurons

**DOI:** 10.3390/ijms26157162

**Published:** 2025-07-24

**Authors:** Viviana Soto-Mercado, Miguel Mendivil-Perez, Carlos Velez-Pardo, Marlene Jimenez-Del-Rio

**Affiliations:** 1 Neuroscience Research Group, Institute of Medical Research, Faculty of Medicine, University Research Headquarters, University of Antioquia, Calle 70#52-21 and Calle 62#52-59, Building 1, Laboratory 411/412, Medellin 050010, Colombia; viviana.soto@udea.edu.co (V.S.-M.); calberto.velez@udea.edu.co (C.V.-P.); 2 Neuroscience Research Group, Faculty of Nursing, University Research Headquarters, University of Antioquia, Calle 62#52-59, Building 1, Laboratory 411/412, Medellin 050010, Colombia; miguel.mendivil@udea.edu.co

**Keywords:** Alzheimer, cholinergic, familial, microglia, mutation, neurons, sporadic, variant

## Abstract

In familial Alzheimer’s disease (FAD), presenilin 1 (PSEN1) E280A cholinergic-like neurons (ChLNs) induce aberrant secretion of extracellular amyloid beta (eAβ). How PSEN1 E280A ChLNs-eAβ affects microglial activity is still unknown. We obtained induced microglia-like cells (iMG) from human peripheral blood cells (hPBCs) in a 15-day differentiation process to investigate the effect of bolus addition of Aβ42, PSEN1 E280A cholinergic-like neuron (ChLN)-derived culture supernatants, and PSEN1 E280A ChLNs on wild type (WT) iMG, PSEN1 E280A iMG, and sporadic Alzheimer’s disease (SAD) iMG. We found that WT iMG cells, when challenged with non-cellular (e.g., lipopolysaccharide, LPS) or cellular (e.g., Aβ42, PSEN1 E280A ChLN-derived culture supernatants) microenvironments, closely resemble primary human microglia in terms of morphology (resembling an “amoeboid-like phenotype”), expression of surface markers (Ionized calcium-binding adapter molecule 1, IBA-1; transmembrane protein 119, TMEM119), phagocytic ability (high pHrodo™ Red *E. coli* BioParticles™ phagocytic activity), immune metabolism (i.e., high generation of reactive oxygen species, ROS), increase in mitochondrial membrane potential (ΔΨm), response to ATP-induced transient intracellular Ca^2+^ influx, cell polarization (cluster of differentiation 68 (CD68)/CD206 ratio: M1 phenotype), cell migration activity according to the scratch wound assay, and especially in their inflammatory response (secretion of cytokine interleukin-6, IL-6; Tumor necrosis factor alpha, TNF-α). We also found that PSEN1 E280A and SAD iMG are physiologically unresponsive to ATP-induced Ca^2+^ influx, have reduced phagocytic activity, and diminished expression of Triggering Receptor Expressed on Myeloid Cells 2 (TREM2) protein, but when co-cultured with PSEN1 E280A ChLNs, iMG shows an increase in pro-inflammatory phenotype (M1) and secretes high levels of cytokines IL-6 and TNF-α. As a result, PSEN1 E280A and SAD iMG induce apoptosis in PSEN1 E280A ChLNs as evidenced by abnormal phosphorylation of protein TAU at residue T205 and cleaved caspase 3 (CC3). Taken together, these results suggest that PSEN1 E280A ChLNs initiate a vicious cycle between damaged neurons and M1 phenotype microglia, resulting in excessive ChLN death. Our findings provide a suitable platform for the exploration of novel therapeutic approaches for the fight against FAD.

## 1. Introduction

Alzheimer’s disease (AD) is a chronic and progressive neurodegenerative disease that occurs as sporadic AD (SAD, onset > 65 years of age) and familial AD (FAD, onset < 64 years of age) [1,2]. SAD and FAD are neuropathologically indistinguishable [3]. Indeed, both disorders are characterized by memory loss due to the loss of cholinergic neurons of the nucleus basalis of Meynert [4,5,6,7,8] projecting into the cortex and hippocampus [9,10], deposition of amyloid plaques formed by extracellular accumulation of amyloid beta 42 (eAβ42), and intracellular accumulation of the abnormally phosphorylated protein TAU [11,12]. In contrast to SAD, FAD is genetically characterized by the presence of mutations in at least three highly penetrant genes: amyloid precursor protein (*APP*, 106 variants), presenilin (*PSEN*) 1 (*PSEN1*, 369 variants), and *PSEN2* (81 variants) (https://www.alzforum.org/mutations, available 7 May 2025), as well as polygenic risk variants [13]. Mutations in the *PSEN* gene, which is the catalytic unit of γ secretase [14], resulted in abnormal metabolism during APP processing that overproduced extracellular eAβ42 [15,16]. Although the precise mechanism by which eAβ42 causes structural and/or functional neuronal deficits remains unclear [17,18], it is widely accepted that eAβ42 induces neuroinflammation—an early event in the neurodegeneration process [19] through TLR4/Rac1/NLRP3 Pathway [20]. Indeed, the neuroinflammatory cascade mediated by primed brain microglia is increasingly recognized as a contributor to the pathogenesis of AD [21]. Therefore, microglia have become an important therapeutic target in AD [22,23].

Microglia are the primary immune cells of the central nervous system (CNS), derived from the myeloid lineage [24]. In humans, microglia are derived from myeloid precursors in the yolk sac and migrate to the brain during early embryonic development, before the formation of the blood–brain barrier. During normal brain development, microglia proliferate and self-renew throughout life, maintaining their population without input from bone marrow-derived macrophages [25]. They play critical roles in immune surveillance, neurogenesis, synaptic pruning, and phagocytosis [26]. Notably, microglia-mediated synaptic loss correlates with cognitive decline in Alzheimer’s disease [27]. In addition, microglia exhibit high plasticity and can be activated into two distinct states in response to environmental stimuli: the pro-inflammatory M1 state and the anti-inflammatory M2 state. The M1 state is induced by stimuli such as lipopolysaccharide (LPS) and results in the release of the pro-inflammatory cytokines interleukin-1 beta (IL-1β), interleukin-6 (IL-6), and tumor necrosis factor alpha (TNF-α), which can lead to neuronal apoptosis and brain damage if hyperactivity persists [28,29]. In addition, several data suggest that IL-6, IL-1β, and TNF-α released by microglia are important biomarkers in Alzheimer’s disease [30]. Thus, reactive microgliosis, characterized by abnormal morphological changes, such as the transition from a ramified to an amoeboid form, increased immunoreactivity for ionized calcium-binding adapter molecule 1 (IBA1), and increased proliferation, is a prominent feature of AD pathology [31]. Importantly, microglia contribute to the phagocytic clearance of Aβ but also to the protection of neuronal tissue. This dual role of microglia in AD pathogenesis highlights their functional complexity [32]. Despite these advances, the lack of appropriate human cellular models, particularly for FAD PSEN1 E280A, has hampered further basic pathophysiological studies linking microglia to brain disease.

Accumulating evidence has shown that alterations in the cholinergic system, which plays a critical role in cognitive functions, such as attention, learning, memory, and motivation [33], are involved in the pathophysiology of AD [34]. Indeed, under normal physiological conditions, neuronal–microglial cholinergic signaling is anti-inflammatory through α7 nicotinic acetylcholine receptors (α7nAChRs) and antioxidant (e.g., by reducing c-Jun N-terminal kinase (JNK) signaling), whereas during chronic neuroinflammation induced by Aβ42 and loss of cholinergic neurons, increased deleterious microglial activation affects hippocampal connectivity, thereby inducing memory loss [35]. Overall, the loss of cholinergic neurons may lead to microglial activation and persistent neuroinflammation, contributing to neurodegeneration [36]. Although it is well established that mutations in PSEN1, including PSEN1 E280A, cause increased Aβ42 deposition, severe cerebellar pathology, and gliosis in postmortem brains [37,38], there are no data to determine whether PSEN1 E280A cholinergic neurons, which secrete Aβ42 [39,40,41], could activate or affect microglial functions in vitro.

Several efforts have been made to obtain microglia to study AD pathology, including animal-based or human induced pluripotent stem cell (hiPSC)-derived microglia [42,43]. However, these methods are time-consuming, technically challenging, and have long differentiation times. Therefore, there is an urgent need to develop efficient and massive cellular models that represent patient-specific microglial responses to FAD. Inspired by the work of Ohgidani and coworkers [44] and others [45,46], we obtained induced microglia-like (iMG) cells from human peripheral blood cells (hPBCs) in a 15-day differentiation process to investigate the effect of bolus addition of Aβ42, PSEN1 E280A cholinergic-like neurons (ChLNs)-derived culture supernatants and PSEN1 E280A ChLNs on WT iMG, PSEN1 E280A iMG, and SAD iMG. Our results indicate that iMG cells closely resemble primary human microglia in terms of morphology, surface markers, phagocytic ability, immune metabolism, polarization, and branching, especially in their inflammatory response to the cellular and non-cellular microenvironments of FAD. Moreover, we found that PSEN1 E280A iMG and SAD iMG are physiologically unresponsive to ATP-induced Ca^2+^ influx and possess reduced phagocytic activity, but when cocultured with PSEN1 E280A ChLNs, iMG shows an increase in pro-inflammatory phenotype (M1) and secreted high amounts of cytokines IL-6 and TNF-α. As a result, PSEN1 E280A iMG and SAD iMG induce apoptosis in PSEN1 E280A ChLNs, as evidenced by abnormal phosphorylation of protein TAU and cleaved caspase (CC3). These interactions instigate a vicious cycle between damaged neurons and M1 phenotypic microglia, resulting in excessive ChLNs death.

## 2. Results

### 2.1. Induced Microglia-like Cells (iMG) from Human Peripheral Blood Mononuclear Cells (hPBMCs) Exhibit a Typical Microglial Phenotype

**Figure 1:**
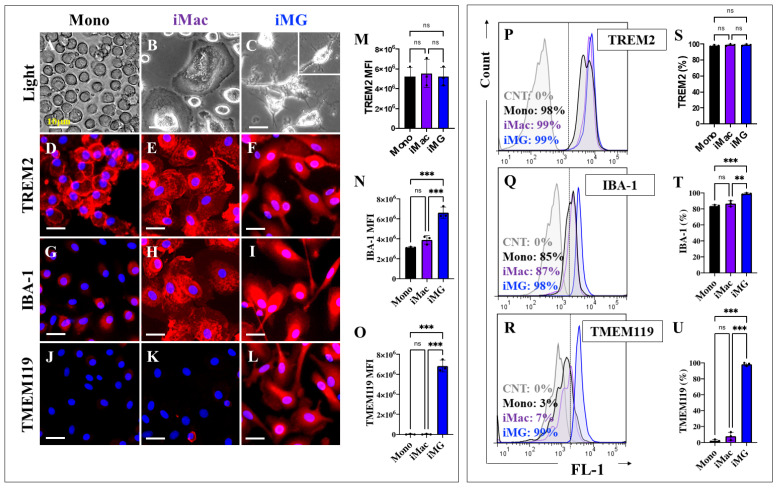
Induced Microglia-like Cells (iMG) exhibit microglial morphological and phenotypic characteristics compared to monocytes (Mono) and induced macrophages (iMac). iMG and iMac cells were induced from human peripheral blood monocytes (Mono) for 15 days. Representative light microscopy images showing Mono (**A**), iMac (**B**), and iMG (**C**) morphology after 15 days. Inset: closed up of a representative iMG cell. Representative fluorescence images showing nuclei (blue) and Triggering Receptor Expressed on Myeloid Cells 2, TREM2 (red) positive Mono (**D**), iMac (**E**), and iMG (**F**). Representative fluorescence images showing nuclei (blue) and Ionized calcium-binding adapter molecule 1, IBA-1 (red)-positive Mono (**G**), iMac (**H**), and iMG (**I**). Representative fluorescence images showing nuclei (blue) and transmembrane protein 119, TMEM119 (red) positive Mono (**J**), iMac (**K**), and iMG (**L**). (**M**) Quantitative analysis of TREM2 mean fluorescence intensity (MFI). (**N**) Quantitative analysis of IBA-1 mean fluorescence intensity (MFI). (**O**) Quantitative analysis of TMEM119 mean fluorescence intensity (MFI). Representative flow cytometry histograms showing TREM2- (**P**), IBA-1- (**Q**), and TMEM119-labeled (**R**) Mono (black bar), iMac (purple bar), and iMG (blue bar). Unstained cells were used as a negative control (gray, CNT). Quantitative analysis of TREM2- (**S**), IBA-1- (**T**), and TMEM119-positive (**U**) cells by flow cytometry analysis. Data are expressed as mean ± SD; ** *p* < 0.01; *** *p* < 0.001; ns = not significant. Photomicrographs, figures/histograms, and bars represent 1 of 3 independent experiments (*n* = 3). Image magnification ×100.

Previously, fresh or frozen PBMC-derived monocytes (Mono) treated with different media containing either Granulocyte-Macrophage Colony-Stimulating Factor (GM-CSF, 10 ng/mL) alone or in combination with IL-34 (100 ng/mL) were shown to generate human-induced macrophage-like cells (iMac) or human-induced microglia-like cells (iMG) [45]. As shown in Figure 1, light microscopy analysis reveals the typical round morphology of mono cells (Figure 1A), the “fried-egg” shape of iMac (Figure 1B), and ramified iMG with a small soma and multiple branching processes (Figure 1C). To further phenotypically characterize the mono, iMac, and iMG, each cell population was analyzed for the presence of the microglia/macrophage/monocyte marker Triggering Receptor Expressed on Myeloid Cells 2, TREM2 [47], the microglia/macrophage marker Ionized calcium-binding adapter molecule 1, IBA-1 [48], and the microglia-specific surface marker transmembrane protein 119, TMEM119 [49]. Indeed, TREM2 was detected in mono (Figure 1D), iMac (Figure 1E), and iMG cells (Figure 1F), with no statistically significant differences in TREM2 expression in intercellular population comparisons (Figure 1M). While no statistically significant differences were found between the expression of IBA-1 in Mono (Figure 1G,N) and iMac (Figure 1H,N), there was a marked difference with the expression of this marker in iMG (Figure 1I,N). TMEM119 analysis shows its almost complete absence in both Mono (Figure 1J,O) and iMac (Figure 1K,O), but it is uniquely expressed in iMG (Figure 1L,O). Similar observations were made using flow cytometry analysis (Figure 1P–U).

### 2.2. Induced Microglia-like Cells **(**iMG) Cells, but Not Monocytes, Respond to ATP-Induced Transient Intracellular Ca^2+^ Influx

**Figure 2:**
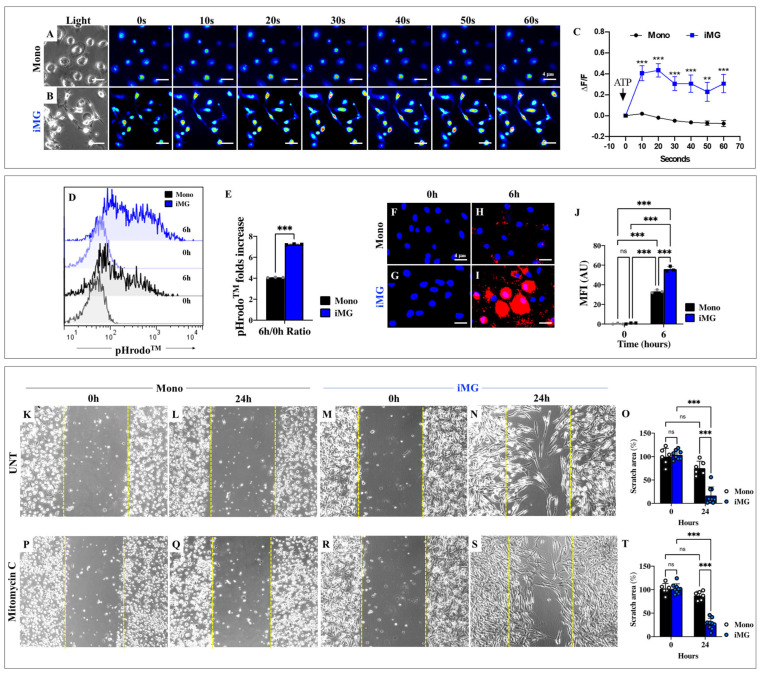
Induced Microglia-like Cells **(**iMG) shows functional response to ATP stimuli, high phagocytic and migratory activity. iMG cells were induced from human peripheral blood monocytes (Mono) for 15 days. Time-lapse images (0, 10, 20, 30, 40, 50, 60 s) of Ca^2+^ fluorescence in Mono (**A**) and iMG (**B**) in response to ATP stimuli. ATP was added to the culture at 0 s (arrow in (**C**)). The Ca^2+^ fluorescence of the cells was then monitored at the indicated times. Color contrast indicates fluorescence intensity: dark blue < light blue < green < yellow < red. (**C**) Normalized mean fluorescence signal (ΔF/F) over time of the cells, indicating temporal cytoplasmic Ca^2+^ increase in response to ATP treatment. (**D**) Representative flow cytometry histogram analysis showing pHrodo^TM^ acidification in Mono (black line) and iMG (blue line) after 0 and 6 h of incubation. (**E**) Quantitative analysis of pHrodo^TM^ mean fluorescence intensity (MFI) ratio (6 h/0 h). Representative fluorescence images and insets showing nuclei and pHrodo^TM^ acidification in mono and iMG after 0 ((**F**,**G**), respectively) and 6 h ((**H**,**I**), respectively) of incubation. Fluorescence images showing nuclei (blue) and Red *E. coli* BioParticles™ (red). (**J**) Quantitative analysis of pHrodo^TM^ mean fluorescence intensity. Representative light microscopy images showing scratch size in untreated (UNT) Mono at 0 h (**K**) and 24 h (**L**) and iMG at 0 h (**M**) and 24 h (**N**). (**O**) Quantitative analysis of scratch size as a percentage of control. Representative light microscopy images showing scratch size in Mono treated with mitomycin C (0.5 mg/mL) for 1 h previous to tissue scratch assay at 0 h (**P**) and 24 h (**Q**) and iMG at 0 h (**R**) and 24 h (**S**). The yellow dashed lines show the boundaries of the scratch wounds. (**T**) Quantitative analysis of scratch size as a percentage of control. Data are expressed as mean ± SD or SEM (calcium analysis). ** *p* < 0.01; *** *p* < 0.001; ns = not significant. Photomicrographs, figures/histograms, and bars represent one of three independent experiments (*n* = 3). Fluorescence image magnification, 100×. Light image magnification, 20×.

We also wanted to characterize iMG functionally. As shown in Figure 2, the response to ATP stimuli was significantly lower in mono (Figure 2A) than in iMG (Figure 2B). Indeed, the average fluorescence change (ΔF/F) was 0.019 ± 0.009, −0.020 ± 0.008, −0.048 ± 0.008, −0.063 ± 0.011, −0.072 ± 0.021, −0.076 ± 0.0029 at 10, 20, 30, 40, 50, and 60 s, respectively (n = 60 mono cells imaged, N = 3 dishes, Figure 2C), whereas ATP stimuli induced a transient increase in intracellular Ca^2+^ in iMG, with an average fluorescence change (ΔF/F) of 0.40 ± 0.061, 0.43 ± 0.075, 0.30 ± 0.066, 0.30 ± 0.083, 0.22 ± 0.091, 0.3 ± 0.088 at 10, 20, 30, 40, 50, and 60 s, respectively (n = 60 iMG cells imaged, N = 3 dishes). Overall, iMG responded to ATP-induced Ca^2+^ influx, whereas Mono did not, resulting in a maximum increase of 23-fold at 20 s compared to Mono (Figure 2C).

### 2.3. Induced Microglia-like Cells (iMG) Show Higher Phagocytic Activity than Monocytes Exposed to pHrodo™ Red E. coli BioParticles™ Conjugate

Microglia are known to have phagocytic activity, for example, towards apoptotic cells, invading microbes, or synapses [50], and this cellular behavior can be recapitulated by quantifying bead uptake over time [51]. Therefore, the pHrodo™ Red *E. coli* BioParticles™ conjugate was used to study phagocytic activity [52,53]. Since the phagocytic index peaks within the first 6 h and then declines, as standardized with glial cells such as astrocyte-like cells in the laboratory, we determined the phagocytic activity of mono and iMG at 0 and 6 h. Flow cytometry analysis shows that both mono and iMG exhibited similar basal levels of pHrodo^TM^ red signal at 0 h (Figure 2D,E); however, after 6 h of incubation, the pHrodo^TM^ red signal was significantly higher in iMG (+80%, 1.77-fold increase) compared to mono (Figure 2D,E). Similar observations were made using fluorescence microscopy (Figure 2F–J).

### 2.4. Scratch Wound Induces Higher Migration Activity in Induced Microglia-like Cells (iMG) Rather than in Monocytes (Mono)

We then examined whether iMG differentiation promoted migration activity. Therefore, a scratch wound assay was used to analyze cell migration [54]. According to image analysis, both Mono and iMG migrated into the cell-free area by 24 h, but the response of iMG was almost +616% higher than Mono in both untreated (Figure 2K–O). To confirm that the migration of the iMG cells was due to differentiation and a natural response to the scratch wound rather than proliferation, the Mono and iMG cells were treated with mitomycin C, an antibiotic and antineoplastic agent. Both the mitomycin C-treated Mono and iMG cells migrated, exhibiting a similar pattern to the untreated cells (Figure 2P,T).

### 2.5. Induced Microglia-like Cells (iMG) Exposed to Lipopolysaccharide (LPS) and Amyloid Beta 42 (Aβ42) Stimuli Generate High Levels of Intracellular Reactive Oxygen Species (ROS) and Increase Mitochondrial Membrane Potential (∆Ψm)

**Figure 3:**
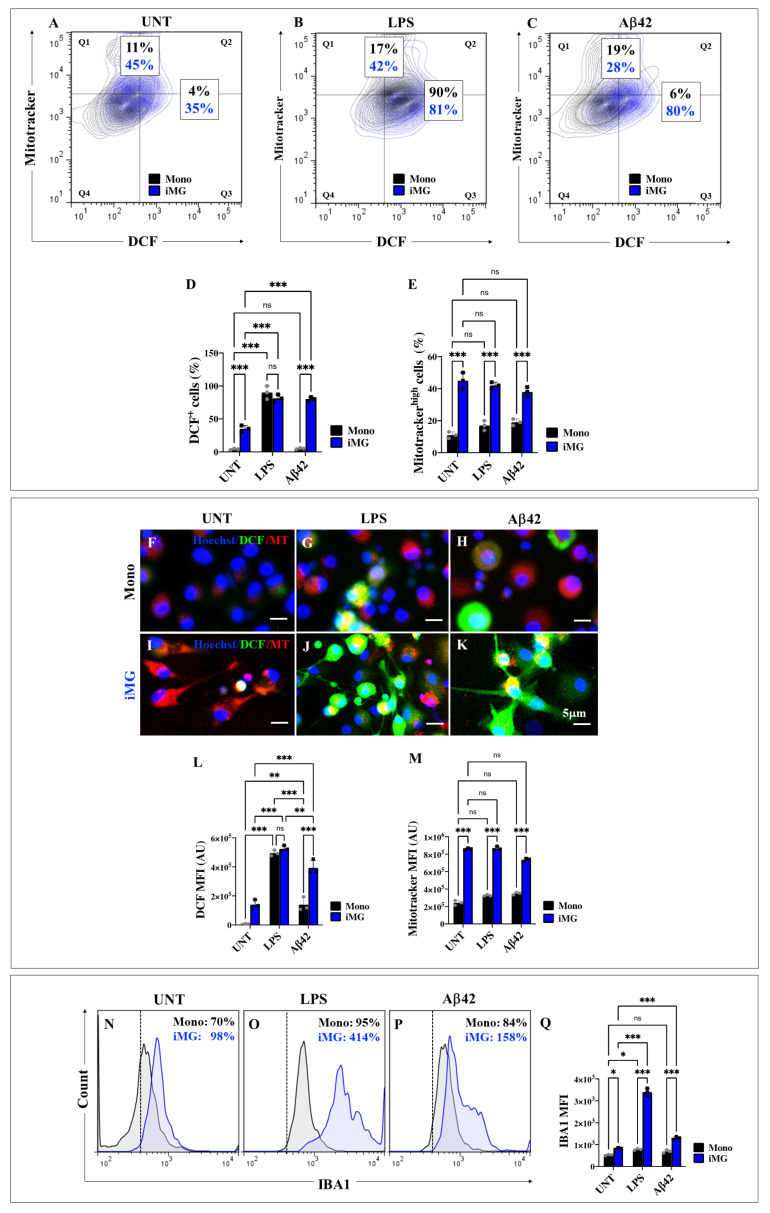
Induced Microglia-like Cells (iMG) show high levels of intracellular reactive oxygen species (ROS), an increase in mitochondrial membrane potential (∆Ψm), and Ionized calcium-binding adapter molecule 1 (IBA-1) reactivity after lipopolysaccharide (LPS) and amyloid-beta 42 (Aβ42) stimuli. iMG cells were induced from human peripheral blood monocytes (Mono) for 15 days. Cells were then left untreated or treated with LPS and Aβ42, as described in Section 4. Representative contour images showing dichlorofluorescein-positive (DCF+) and MitoTracker^®^ high mono (black contour) and iMG (blue contour) in untreated conditions (**A**) or after LPS (**B**) and Aβ42 (**C**) stimulation. (**D**) Quantitative analysis of DCF+ cells (quadrants Q2 + Q3). (**E**) Quantitative analysis of MitoTracker^®^ high cells (quadrants Q1 + Q2). Representative fluorescence images showing Hoechst (blue)/DCF (green)/MitoTracker (MT; red) positive staining in untreated (**F**) or LPS-treated (**G**) and Aβ42-treated mono (**H**); and untreated (**I**) or LPS-treated (**J**) and Aβ42-treated iMG (**K**). (**L**) Quantitative analysis of DCF mean fluorescence intensity (MFI). (**M**) Quantitative analysis of MitoTracker mean fluorescence intensity (MFI). Representative flow cytometry histograms showing IBA-1-positive staining in untreated Mono (black line) and iMG (blue line) (**N**) or in LPS-treated Mono (black line) and iMG (blue line) (**O**) and Aβ42-treated Mono (black line) and iMG (blue line) (**P**). (**Q**) Quantitative analysis of IBA-1 reactivity. Data are expressed as mean ± SD. * *p* < 0.05; ** *p* < 0.01; *** *p* < 0.001; ns = not significant. Photomicrographs, figures/histograms, and bars represent one of three independent experiments (*n* = 3). Image magnification, 100×.

To evaluate whether the potent stimulator of innate or natural immunity, LPS, and the Alzheimer’s disease-related Aβ42 peptide induced ROS generation and/or exhibited changes in ΔΨm in iMG cells, Mono (included as control) and iMG cells were left untreated or treated with LPS (100 μg/mL) or Aβ42 peptide (10 μg/mL) for 24 h. As shown in Figure 3, untreated iMG cells exhibited higher levels of ROS generation (+775%) than Mono (Figure 3A,D). However, when cells were exposed to LPS, ROS production increased to similar levels in both cells (Figure 3B,D). Interestingly, only iMG exposed to Aβ42 peptide stimuli increased ROS generation by +1233% (Figure 3C,D). Similarly, untreated iMG showed higher levels of ∆Ψm (+309%, Figure 3A,E) than those treated with LPS (+147%, Figure 3B,E) or Aβ42 peptide (+47%, Figure 3C,E) stimuli than Mono (Figure 3A–C,E). Similar results were obtained by fluorescence microscopy (Figure 3F–M).

### 2.6. Lipopolysaccharide (LPS) or Amyloid Beta 42 (Aβ42) Peptide Stimuli Increase the Expression Levels of Ionized Calcium-Binding Adapter Molecule 1 (IBA1) in Induced Microglia-like Cells (iMG) Rather than in Monocytes (Mono)

IBA1 is the most widely used marker for immunohistochemical analysis of microglia and is often characterized as a typical microglial activation marker in AD pathology [55]. Therefore, we investigated whether iMG or Mono cells express IBA1 under LPS or Aβ42 stimulation. Flow cytometry analysis shows that untreated iMG had higher basal levels of IBA1 than Mono (+55%, Figure 3N,Q). When cells were exposed to LPS or Aβ42, IBA1 reactivity in iMG increased significantly by +322% (Figure 3O,Q) and +61% (Figure 3P,Q), respectively, compared to untreated iMG (Figure 3N), but IBA1 reactivity in Mono treated with LPS increased slightly by +36% (Figure 3O,Q) and Aβ42 by +20% (Figure 3P,Q) compared to untreated Mono (Figure 3N). Overall, IBA1 reactivity was significantly higher in iMG than in Mono under all conditions tested (Figure 3N–Q).

### 2.7. Lipopolysaccharide (LPS) or Amyloid Beta 42 (Aβ42) Peptide Stimuli Increase the Internal Granularity of Cells (Area) and Expression Levels of Actin in Induced Microglia-like Cells (iMG)

**Figure 4:**
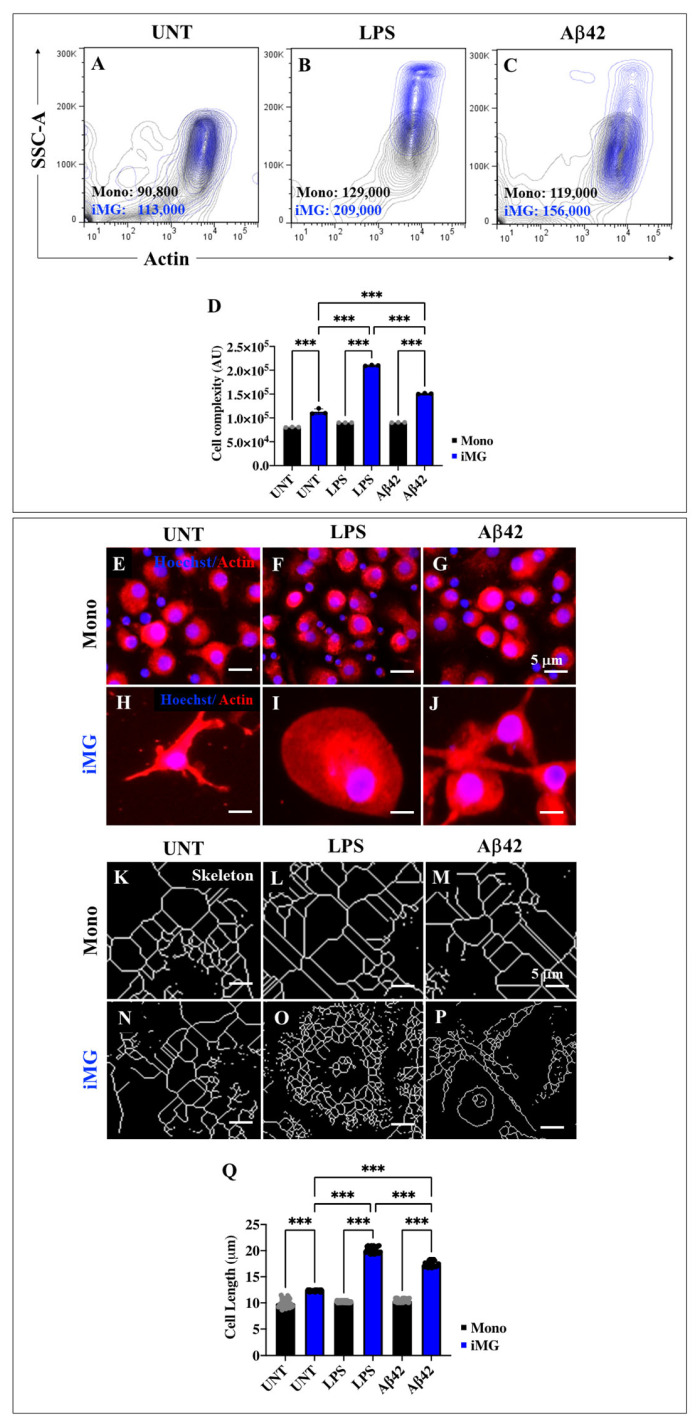
Induced Microglia-like Cells (iMG) show high changes in cell complexity after lipopolysaccharide (LPS) and amyloid-beta 42 (Aβ42) stimuli. iMG cells were induced from human peripheral blood monocytes (Mono) for 15 days. Cells were then left untreated or treated with LPS and Aβ42, as described in the Section 4. Representative contour images showing actin (*x*-axis) and Side Scatter Area, SSC-A (*y*-axis) double analysis of Mono (black contour) and iMG (blue contour) in untreated conditions (**A**) or after LPS (**B**) and Aβ42 (**C**) stimulation. (**D**) Quantitative analysis of actin-labeled cell complexity mean fluorescent intensity, MFI (SSC-A). Representative fluorescence images showing Hoechst (blue)/actin (red) positive staining in untreated (**E**) or LPS-treated (**F**) and Aβ42-treated mono (**G**); and untreated (**H**) or LPS-treated (**I**) and Aβ42-treated iMG (**J**). Representative skeleton images generated from actin-stained cells in untreated (**K**) or LPS-treated (**L**) and Aβ42-treated Mono (**M**); and untreated (**N**) or LPS-treated (**O**) and Aβ42-treated iMG (**P**). (**Q**) Quantitative analysis of cell length from skeleton analyses. Data are expressed as mean ± SD. *** *p* < 0.001. Micrographs, figures, and bars represent one of three independent experiments (*n* = 3). Image magnification, 100×.

Healthy (homeostatic) microglia are known to have multiple ramifications, small, spherical somata, and symmetrically distributed, thin, highly branched processes. In response to injury or neurotoxic stimuli, these cells are associated with an increase in cell body size and a decrease in its roundness, reflecting the morphological transition from a ramified ‘quiescent’ to an activated amoeboid-like phenotype [56]. Overall, microglia undergo a morphological and functional transformation upon neuronal insult. This phenomenon can be evidenced by changes in the cellular disposition of actin, skeleton length, and/or cell complexity [57]. Therefore, we first evaluated side scatter (SSC) area (SSC-A) and actin expression in both Mono and iMG cells in the presence or absence of LPS or Aβ42 stimuli. Figure 4 shows that untreated iMG cells exhibited more internal granularity (Figure 4A) compared to untreated Mono cells (Figure 4A,D). After exposure to LPS or Aβ42, the granularity of Mono cells did not change significantly compared to untreated Mono (Figure 4B–D). In contrast, iMG exposed to either LPS or Aβ42 increased cell complexity by +85% (Figure 4B,D) and +38% (Figure 4C,D), respectively. Fluorescence microscopy revealed that neither LPS nor Aβ42 altered the distribution of cellular actin in Mono (Figure 4F,G), which showed a rounded morphology similar to untreated Mono cells (Figure 4E). In contrast, LPS or Aβ42 induced a dramatic morphological change in iMG, from a ramified phenotype (Figure 4H) to an amoeboid-like (Figure 4I) or reactive phenotype (Figure 4J). Analysis of the skeleton length shows that it remained unchanged in untreated Mono cells (Figure 4K) or Mono cells treated with LPS (Figure 4L) or Aβ42 (Figure 4M,Q). We found that the skeleton length in untreated iMG (Figure 4N) increased by +24% compared to Mono (Figure 4K). When iMG cells were treated with LPS and Aβ42, the skeleton length increased by +100% and +67%, respectively (Figure 4Q). Overall, the skeletal length of processes was significantly higher in iMG than in Mono under all conditions tested (Figure 4Q), but LPS induced a more pronounced increase in skeletal length than Aβ42 when exposed to iMG (Figure 4Q).

### 2.8. Lipopolysaccharide (LPS) or Amyloid Beta 42 (Aβ42) Stimuli Induce the Release of Pro-Inflammatory Cytokines Interleukin-6 (IL-6) and Tumor Necrosis Factor Alpha (TNF-α), but Anti-Inflammatory IL-10 Is Absent in Induced Microglia-like Cells (iMG)

**Figure 5:**
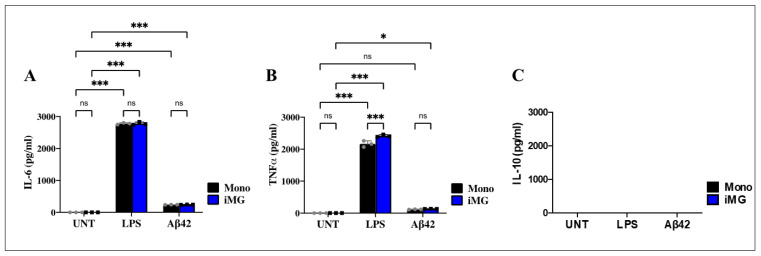
Induced Microglia-like Cells (iMG) increase interleukin-6 (IL-6) and Tumor necrosis factor alpha (TNF-α), but IL-10 release does not increase after lipopolysaccharide (LPS) and amyloid-beta 42 (Aβ42) stimulation. iMG cells were induced from human peripheral blood monocytes (Mono) for 15 days. The cells were then left untreated or treated with LPS and Aβ42, as described in the Section 4. Quantitative analysis of IL-6 (**A**), TNF-α (**B**), and IL-10 (**C**) release in Mono (black bar) and iMG (blue bar) untreated or treated with LPS or Aβ42 for 24 h. Data are presented as means ± SD. * *p* < 0.05; *** *p* < 0.001; ns = not significant. Bars represent one of three independent experiments (*n* = 3).

Both LPS and Aβ42 are known to induce M1 polarization [58]. Accordingly, we sought to evaluate the cytokine release profile in Mono and iMG treated with LPS or Aβ42 peptide for 24 h. Cytometric Bead Array (CBA) of human inflammatory cytokines assay shows that LPS or Aβ42 induced detectable levels of IL-6 (Figure 5A) and TNF-α (Figure 5B), but no detectable IL-10 (Figure 5C) was found in Mono and iMG cells. In fact, while LPS induced the release of IL-6 equally in Mono and iMG cells (~2780 pg/mL; Figure 5A), Aβ42 induced the release of IL-6 at low levels in Mono and iMG cells (~240 pg/mL; Figure 5A). Overall, LPS increased the release of IL-6 in iMG/Mono by +1058% compared to Aβ42. Similarly, LPS was much more effective in inducing TNF-α release in Mono and iMG than Aβ42 (Figure 5B); however, LPS significantly increased the release of TNF-α in iMG compared to Mono cells.

### 2.9. Presenilin 1 (PSEN1) E280A Cholinergic-like Neuron (ChLN) Culture Supernatant Induces High Intracellular Levels of Reactive Oxygen Species (ROS) and Increases Mitochondrial Membrane Potential (∆Ψm) in Wild Type (WT) Induced Microglia-like Cells (iMG)

**Figure 6:**
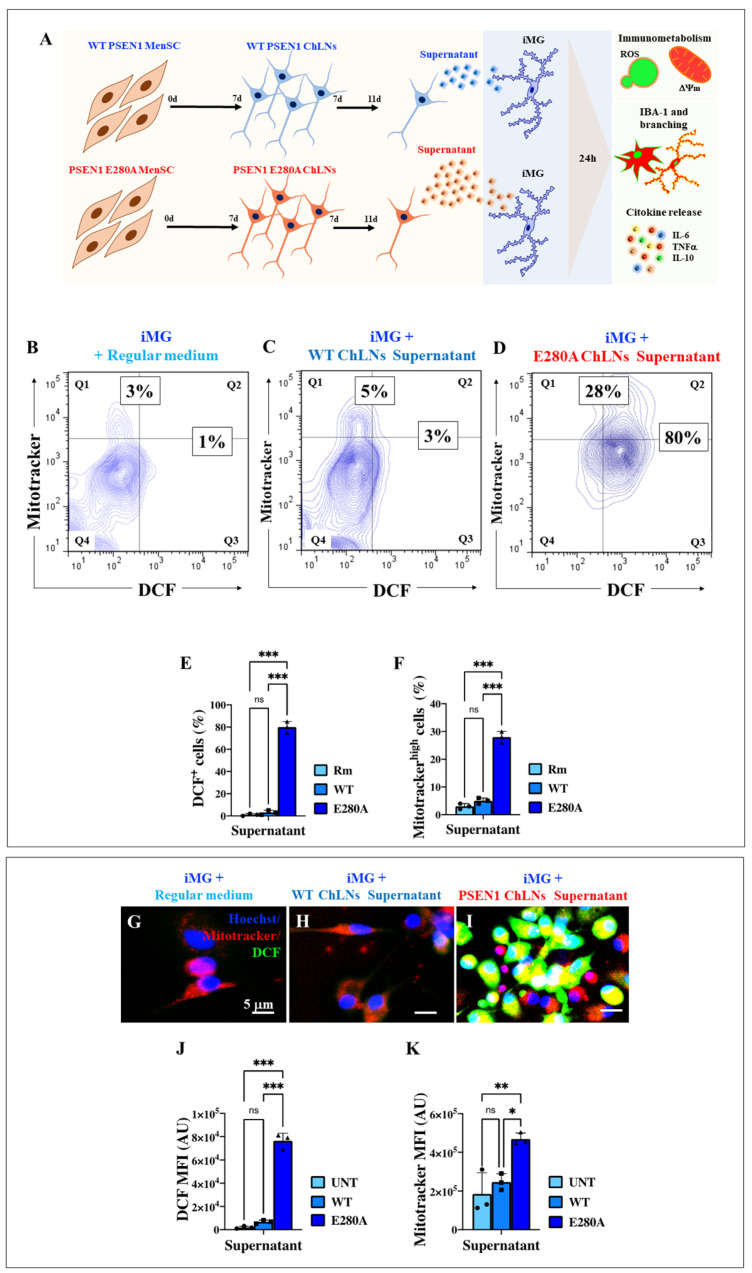
Induced Microglia-like Cells (iMG) show high levels of intracellular reactive oxygen species (ROS) and an increase in mitochondrial membrane potential (∆Ψm) after stimulation with cholinergic supernatants. iMG cells were induced from human peripheral blood monocytes (Mono) for 15 days. Subsequently, cells were left untreated or treated with wild type (WT) presenilin 1 (PSEN1) and PSEN1 E280A ChLNs-derived supernatants, as described in the Section 4. Schematic representation of the methodological procedure and comparative analyses (**A**). Representative contour images showing dichlorofluorescein-positive (DCF+) and MitoTracker in WT iMG (blue contour) treated with regular medium (**B**) or WT PSEN1 ChLNs- (**C**) and PSEN1 E280A ChLNs-derived supernatants (**D**). Quantitative analysis of DCF+ cells (quadrants Q2 + Q3) (**E**). Quantitative analysis of MitoTracker high cells (quadrants Q1 + Q2) (**F**). Representative fluorescence images showing Hoechst (blue)/DCF (green)/MitoTracker (MT; red) positive staining of iMG treated with regular medium (**G**) or WT PSEN1 ChLNs (**H**) and PSEN1 E280A ChLN-derived supernatants (**I**). Quantitative analysis of DCF mean fluorescence intensity (MFI) (**J**). Quantitative analysis of MitoTracker MFI (**K**). Data are expressed as mean ± SD. * *p* < 0.05; ** *p* < 0.01; *** *p* < 0.001; ns = not significant. Micrographs, figures, and bars represent one of three independent experiments (*n* = 3). Image magnification, 100×.

Previously, PSEN1 E280A ChLNs were shown to secrete high levels of Aβ42 into the culture medium [41,59]. This observation prompted us to first investigate whether the supernatant from cultured WT ChLNs or PSEN1 E280A ChLNs induce ROS generation and mitochondrial damage in WT iMG (Figure 6A). WT iMG cells left in regular culture medium (Figure 6B) or exposed to supernatants derived from WT ChLNs (Figure 6C) showed similar low basal levels of ROS generation and ΔΨm (Figure 6B,C,E,F). In contrast, WT iMG cells exposed to PSEN1 E280A ChLNs-derived supernatant significantly increased ROS production by +7900% and provoked an increase in ΔΨm by +833% (Figure 6D–F) compared to untreated iMG. Similar results were obtained by fluorescence microscopy analysis (Figure 6G–K).

### 2.10. Presenilin 1 (PSEN1) E280A Cholinergic-like Neuron (ChLN) Culture Supernatant Stimulates High Levels of Ionized Calcium-Binding Adapter Molecule 1 (IBA1) Expression and Increases the Elongation Process in Wild Type (WT) Induced Microglia-like Cells (iMG)

**Figure 7:**
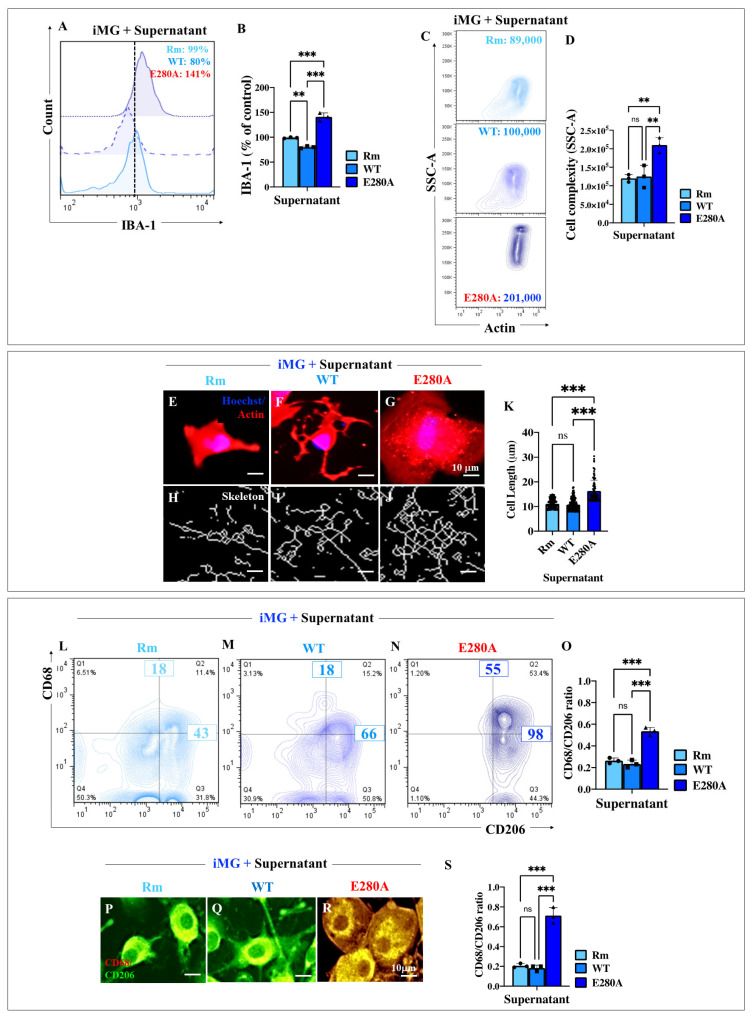
Induced Microglia-like Cells (iMG) show high changes in cell complexity and inflammatory profile after stimulation with familial Alzheimer’s disease (FAD) cholinergic culture supernatants. iMG cells were induced from human peripheral blood monocytes (Mono) for 15 days. Cells were then left untreated or treated with wild type (WT) and presenilin 1 (PSEN1) E280A cholinergic-like neurons (ChLNs)-derived supernatants, as described in the Section 4. (**A**) Representative flow cytometry histograms showing Ionized calcium-binding adapter molecule 1, IBA-1 positive staining in iMG treated with regular medium (cyan blue line) or WT ChLNs (aqua blue line) and PSEN1 E280A ChLN-derived supernatants (cobalt blue line). (**B**) Quantitative analysis of IBA-1 reactivity. (**C**) Representative contour images showing actin (*x*-axis) and Side Scatter Area (SSC-A, *y*-axis) double analysis of iMG treated with regular medium (cyan blue contour) or WT ChLNs (aqua blue contour) and PSEN1 E280A ChLNs-derived supernatants (cobalt blue contour). (**D**) Quantitative analysis of actin-labeled iMG complexity mean fluorescent intensity mean fluorescent intensity, MFI (Side Scatter Area, SSC-A). Representative fluorescence images showing Hoechst (blue)/actin (red) positive staining in iMG treated with regular medium (**E**) or WT ChLNs (**F**) and PSEN1 E280A ChLNs-derived supernatants (**G**). Representative skeleton images generated from actin-stained iMG treated with regular medium (**H**) or WT ChLNs (**I**) and PSEN1 E280A ChLNs-derived supernatants (**J**). Quantitative analysis of cell length from skeleton analyses (**K**). Representative contour images showing cluster of differentiation 206 (CD206, *x*-axis) and CD68 (*y*-axis) double analysis in iMG treated with regular medium (cyan blue contour; (**L**)) or WT ChLNs (aqua blue contour; (**M**)) and PSEN1 E280A ChLNs-derived supernatants (cobalt blue contour; (**N**)). Quantitative analysis of CD68/CD206 ratio (quadrants Q1 + Q2/quadrants Q2 + Q3) (**O**). Representative fluorescence images showing CD68 (red)/CD206 (green) positive staining in iMG treated with regular medium (**P**) or WT ChLNs (**Q**) and PSEN1 E280A ChLNs-derived supernatants (**R**). Quantitative analysis of the CD68/CD206 ratio calculated from CD68/CD206 MFI (**S**). Data are expressed as mean ± SD. ** *p* < 0.01; *** *p* < 0.001; ns = not significant. The photomicrographs, figures, and bars represent one out of three independent experiments (*n* = 3). Image magnification, 100×.

We next examined whether culture-derived supernatants induced cell complexity and/or IBA1 expression in WT iMG. Figure 7 shows that iMG exposed to either Rm- or WT ChLNs-derived culture supernatants did not induce changes in IBA1 expression (Figure 7A,B) or cell complexity (Figure 7C,D). On the contrary, iMG exposed to PSEN1 E280A ChLNs-derived culture supernatants increased IBA1 expression by +76% (Figure 7A,B) and cell complexity by +101% (Figure 7A,B) compared to iMG treated with WT ChLNs-derived supernatants (Figure 7A–D). Notably, iMG under PSEN1 E280A ChLNs-derived supernatant showed obvious morphological changes characterized by multiple branching processes associated with an increase in expression and reorganization of actin proteins (Figure 7G) compared to iMG under Rm (Figure 7E) or iMG exposed to WT ChLNs-derived supernatant (Figure 7F). Moreover, Rm condition (Figure 7H,K) and WT ChLNs-derived supernatant (Figure 7I,K) show similar skeleton lengths; in contrast, iMG exposed to PSEN1 E280A ChLNs-derived supernatant increased the total length of processes by +51% (Figure 7J,K) compared to WT ChLNs-derived supernatant.

### 2.11. Presenilin 1 (PSEN1) E280A Cholinergic-like Neuron (ChLN) Culture Supernatant Stimulates High Expression of Pro-Inflammatory Cluster of Differentiation 68 (CD68) in Wild Type (WT) Induced Microglia-like Cells (iMG)

It has been established that microglia can polarize into either the M1 pro-inflammatory (e.g., CD68-positive) or M2 anti-inflammatory (e.g., CD206-positive) phenotype in response to various microenvironmental perturbations [60]. We therefore investigated whether PSEN1 E280A ChLNs supernatant alters the CD68/CD206 phenotype in WT iMG. Indeed, surface flow cytometry analysis shows that WT iMG cells did not change the CD68/CD206 ratio when exposed to either Rm (Figure 7L,O) or WT ChLNs supernatant (Figure 7M,O), but the ratio increased in favor of CD68 by almost 3-fold in iMG exposed to PSEN1 E280A ChLNs supernatant (Figure 7N,O). A similar CD68/CD206 phenotypic profile was confirmed by immunofluorescence microscopy analysis (Figure 7P–S).

### 2.12. Presenilin 1 (PSEN1) E280A Cholinergic-like Neuron (ChLN) Culture Supernatant Induces the Release of Cytokine Interleukin-6 (IL-6) and Tumor Necrosis Factor Alpha (TNF-α) in Induced Microglia-like Cells (iMG)

**Figure 8:**
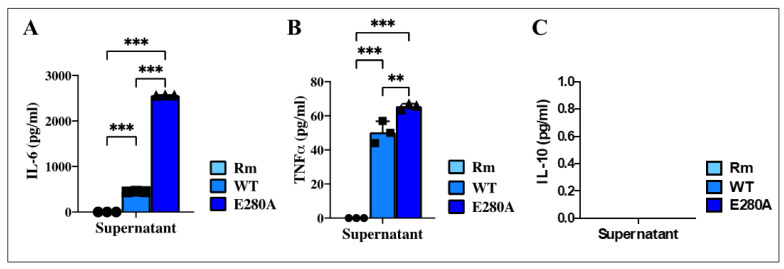
Induced Microglia-like Cells (iMG) increase interleukin-6 (IL-6) and Tumor necrosis factor alpha (TNF-α), but IL-10 release does not increase after Alzheimer’s disease cholinergic-like neuron (ChLN) supernatant stimuli. iMG cells were induced from human peripheral blood monocytes (Mono) for 15 days. Subsequently, cells were left untreated (Rm, cyan blue bar) or treated with WT (aqua blue bar) and PSEN1 E280A ChLNs (cobalt blue bar)-derived supernatants, as described in the Section 4. Quantitative analysis of IL-6 (**A**), TNF-α (**B**), and IL-10 (**C**) release after 24 h. Data are presented as means ± SD. ** *p* < 0.01; *** *p* < 0.001. Bars represent one of three independent experiments (*n* = 3).

Further analysis of iMG cells showed that the supernatants had a differential effect on cytokine secretion. As shown in Figure 8A, iMG treated with Rm secreted undetectable or no IL-6 after 24 h of exposure, whereas iMG treated with either WT or PSEN1 E280A ChLNs-derived supernatant significantly increased IL-6 secretion, albeit at different levels. While iMG exposed to WT ChLNs-derived supernatant secreted 448 pg/mL, iMG exposed to PSEN1 E280A ChLNs-derived supernatant secreted 2570 pg/mL. Overall, iMG increased IL-6 secretion by +474% when treated with PSEN1 E280A ChLNs-derived supernatant compared to WT ChLNs-derived supernatant (Figure 8A). Similar to the IL-6 secretion profile, iMG treated with Rm secreted undetectable or almost no TNF-α, whereas iMG secreted 50 pg/mL and 66 pg/mL when treated with WT or PSEN1 E280A ChLNs-derived supernatant, respectively. Notably, PSEN1 E280A supernatant significantly increased TNF-α secretion (+32%) in iMG compared to iMG treated with WT supernatant (Figure 8B). Analysis of IL-10 secretion shows that IL-10 was not detectable under the present experimental conditions (Figure 8C).

### 2.13. Presenilin 1 (PSEN1) E280A and Sporadic AD (SAD) Induced Microglia-like Cells (iMG) Are Unresponsive to ATP Stimulation

**Figure 9:**
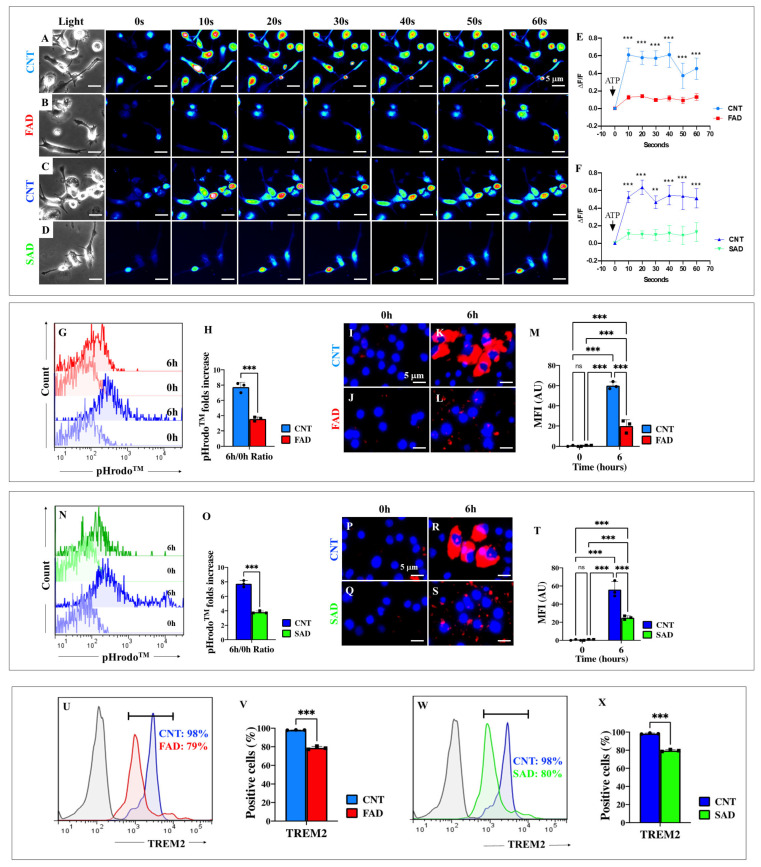
Familial Alzheimer’s disease (FAD, presenilin1 (PSEN1) E280A) and sporadic Alzheimer’s disease (SAD) induced microglia-like cells (iMG) display dysfunctional response to ATP and low phagocytic activity. iMG cells were induced for 15 days from peripheral blood monocytes obtained from FAD and SAD patients or age- and sex-matched controls. Time-lapse images (0, 10, 20, 30, 40, 50, 60 s) of Ca^2+^ fluorescence in control (**A**,**C**), FAD (**B**), and SAD iMG (**D**) in response to ATP stimuli. ATP was added to the culture at 0 s (arrow in (**E**,**F**)). The Ca^2^⁺ fluorescence of the cells was then monitored at the indicated times. Color contrast indicates fluorescence intensity: dark blue < light blue < green < yellow < red. (**E**) Normalized mean fluorescence signal (ΔF/F) over time of age/sex-matched iMG control (cyan blue line) and FAD iMG (red line) showing temporal cytoplasmic Ca^2+^ elevation in response to ATP treatment. (**F**) Normalized mean fluorescence signal (ΔF/F) over time of age/sex-matched iMG control (cobalt blue line) and SAD iMG (green line) indicating temporal cytoplasmic Ca^2+^ elevation in response to ATP treatment. (**G**) Representative flow cytometry histogram analysis showing pHrodoTM acidification in age/sex matched iMG control (cyan blue histogram) and FAD iMG (red histogram) after 0 and 6 h of incubation. (**H**) Quantitative analysis of pHrodoTM mean fluorescence intensity (MFI) ratio (6 h/0 h). Representative fluorescence images showing nuclei and pHrodoTM acidification in age/sex-matched iMG control and FAD iMG after 0 (**I**,**J**) and 6 (**K**,**L**) hours of incubation. (**M**) Quantitative analysis of pHrodoTM mean fluorescence intensity. (**N**) Representative flow cytometry histogram analysis showing pHrodoTM acidification in age/sex-matched iMG control (cobalt blue histogram) and SAD iMG (green histogram) after 0 and 6 h of incubation. (**O**) Quantitative analysis of pHrodoTM mean fluorescence intensity (MFI) ratio (6 h/0 h). Representative fluorescence images showing nuclei and pHrodoTM acidification in age/sex matched iMG control and SAD iMG after 0 (**P**,**Q**), respectively) and 6 (**R**,**S**), respectively) hours of incubation. (**T**) Quantitative analysis of pHrodo^TM^ mean fluorescence intensity. (**U**) Representative flow cytometry histograms showing expression of TREM2 protein in WT (cyan blue) and FAD iMG (red). Unstained cells were used as a negative control (gray, CNT). (**V**) Quantitative analysis of TREM2-positive cells (%). (**W**) Representative flow cytometry histograms showing expression of TREM2 protein in WT (cobalt blue) and SAD iMG (green). Unstained cells were used as a negative control (gray, CNT). (**X**) Quantitative analysis of TREM2-positive cells (%). Data are expressed as mean ± SD or SEM (calcium analysis). ** *p* < 0.01; *** *p* < 0.001; ns = not significant. Photomicrographs, figures/histograms, and bars represent one of three independent experiments (*n* = 3). Fluorescence image magnification, 100×. Light image magnification, 100×.

We sought to determine whether SAD or mutant iMG responded differently to ATP stimuli. As shown in Figure 9, WT iMG (Figure 9A,C) responded similarly to ATP-induced Ca^2+^ influx, with a maximal mean fluorescence change (ΔF/F) of 0.61 ± 0.14 (Figure 9E) and 0.54 ± 0.10 (Figure 9F) at 40 s, respectively (n = 60 iMG cells imaged, *N* = 3 dishes). In contrast, neither PSEN1 E280A (ΔF/F = 0.12 ± 0.03; Figure 9B,E) nor SAD iMG (ΔF/F = 0.11 ± 0.03; Figure 9D,F) responded to ATP stimuli. Overall, mutant and SAD iMG had a ~5-fold reduced ATP-induced Ca^2+^ influx response compared to WT iMG cells (Figure 9E,F).

### 2.14. Presenilin 1 (PSEN1) E280A and Sporadic AD (SAD) Induced Microglia-like Cells (iMG) Show Defective Phagocytosis Due to Diminished Expression of Triggering Receptor Expressed on Myeloid Cells 2 (TREM2)

Analysis of phagocytosis showed that mutant (Figure 9G) and SAD (Figure 9N) iMG exhibited 2.17-fold reduced phagocytic activity compared to WT iMG according to the pHrodo^TM^ assay (Figure 9H,O). Similar observations were made using immunofluorescence microscopy analysis (Figure 9I–M,P–T). Interestingly, TREM 2 protein expression was reduced by −18% in PSEN1 E280A (Figure 9U) and SAD (Figure 9W) iMG compared to WT iMG (Figure 9V,X).

### 2.15. Presenilin 1 (PSEN1) E280A Cholinergic-like Neurons (ChLNs) in Co-Culture with Wild Type (WT) Induced Microglia-like Cells (iMG) Induce High Levels of Microglial Lineage Marker Transmembrane Protein 119 (TMEM119) and Cellular Complexity

**Figure 10:**
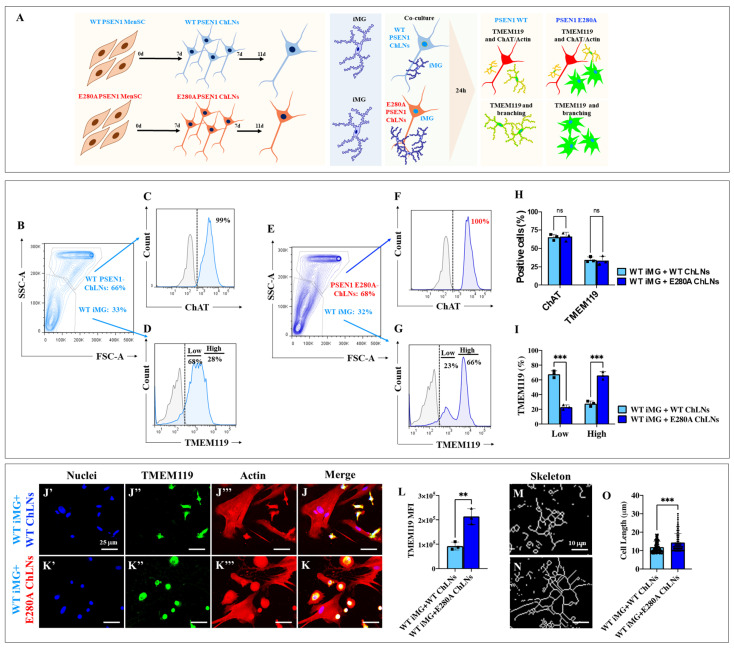
Induced Microglia-like Cells (iMG) show high levels of transmembrane protein 119 (TMEM119) and reactivity in co-culture with cholinergic-like neurons (ChLNs). iMG cells were induced from human peripheral blood monocytes (Mono) for 15 days. The cells were then co-cultured with wild type (WT) ChLNs and presenilin 1 (PSEN1) E280A ChLNs for 24 h, as described in the Section 4. (**A**) Schematic representation of the methodological procedure and comparative analyses. (**B**) Representative flow cytometry analysis showing Forward Scatter Area (FSC-A)/Side Scatter Are (SSC-A) contour images gating iMG and WT ChLNs populations. (**C**) Representative histogram showing the choline acetyltransferase (ChAT)-positive cells from the WT ChLNs population gated in B. (**D**) Representative histogram showing the TMEM119-positive cells from the iMG population gated in B. (**E**) Representative flow cytometry analysis showing FSC-A/SSC-A contour images gating WT iMG (cyan blue) and PSEN1 E280A ChLNs (cobalt blue) populations. (**F**) Representative histogram showing ChAT-positive cells from the PSEN1 E280A ChLNs population gated in E. (**G**) Representative histogram showing TMEM119-positive cells from the WT iMG population gated in E. (**H**) Quantitative analysis of ChAT- and TMEM119-positive cells. (**I**) Quantitative analysis of TMEM119-low and TMEM119-high iMG. Representative fluorescence images showing Hoechst (blue; (**J’**,**K’**))/TMEM119 (green; (**J’’**,**K’’**))/actin (red; (**J’’’**,**K’’’**)) and merge (**J**,**K**) positive staining in iMG co-cultured for 24 h with WT PSEN1 (**J’**–**J’’’**,**J**) and PSEN1 E280A (**K’**–**K’’’**,**K**) ChLNs. (**L**) Quantitative analysis of TMEM119 mean fluorescence intensity (MFI). Representative skeleton images generated from actin-stained iMG co-cultured with WT (**M**) and PSEN1 E280A ChLNs (**N**). (**O**) Quantitative analysis of cell length from skeleton analyses. Data are presented as mean ± SD. ** *p* < 0.01; *** *p* < 0.001; ns = not significant. Micrographs, figures, and bars represent one of three independent experiments (*n* = 3). Image magnification, 40×.

To further characterize mutant and SAD iMG, microglial cells were co-cultured with either WT ChLNs or PSEN1 E280A ChLNs according to the experimental design shown in Figure 10A and described in the Section 4. We first determined the different percentages of cell population per co-culture. As expected, flow cytometry analysis shows two distinct populations in iMG and WT or PSEN1 E280A: a cholinergic population composed of ChLNs (~66–68%) and a microglial population composed of iMG (33–32%) according to Side Scatter Area (SSC-A) and FCS-A analysis (Figure 10B,E), of which almost 100% were positive for the specific cholinergic lineage marker ChAT (Figure 10C,F,H), whereas the microglial marker TMEM119 was expressed at a higher percentage in mutant iMG (66%, Figure 10G,I) than in WT iMG (28%, Figure 10D,I). Immunofluorescence microscopy analysis confirmed these observations (Figure 10J–L). Furthermore, skeleton length analysis revealed that PSEN1 E280A ChLNs induced a +12% change in skeleton length of iMG (Figure 10N,O) compared to the length induced by WT ChLNs in iMG (Figure 10M).

### 2.16. Presenilin 1 (PSEN1) E280A Cholinergic-like Neurons (ChLNs) Induce High Surface Expression of Pro-Inflammatory Cluster of Differentiation 68 (CD68) and Secretion of Cytokine Interleukin-6 (IL-6) and Tumor Necrosis Factor Alpha (TNF-α), but Reduce the Amounts of Secreted Anti-Inflammatory Cytokine IL-10 in Wild Type (WT) Induced Microglia-like Cells (iMG)

**Figure 11:**
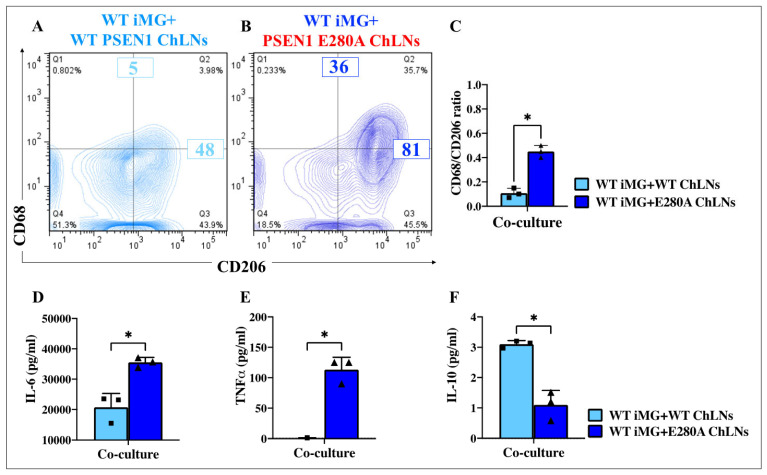
Induced Microglia-like Cells (iMG) show an inflammatory profile and increases interleukin-6 (IL-6) and Tumor necrosis factor alpha (TNF-α) but has a modest release of IL-10 after co-culture with presenilin 1 (PSEN1) E280A cholinergic-like neurons (ChLNs). iMG cells were induced from human peripheral blood monocytes (Mono) for 15 days. The cells were then co-cultured with wild type (WT) PSEN1 (cyan blue contour) and PSEN1 E280A ChLNs (cobalt blue contour) for 24 h, as described in the Section 4. Representative contour images showing double analysis of cluster of differentiation 206 (CD206, *x*-axis) and CD68 (*y*-axis) in iMG co-cultured with WT ChLNs (**A**) and PSEN1 E280A (**B**) ChLNs. (**C**) Quantitative analysis of CD68/CD206 ratio (quadrants Q1 + Q2/quadrants Q2 + Q3). Quantitative analysis of IL-6 (**D**), TNF-α (**E**), and IL-10 (**F**) release after 24 h. Data are presented as means ± SD. * *p* < 0.05. Figures and bars represent one of three independent experiments (*n* = 3).

We also wanted to determine whether PSEN1 E280A ChLNs induce changes in the surface expression of CD68 (M1)/CD206 (M2) and whether they induce the release of cytokines IL-6, IL-10, and TNF-α in iMG. Figure 11 shows that WT ChLNs do not induce significant changes in the expression of CD68 (Figure 11A, Q1, 0.802%) and CD206 (Q3, 43.9%) or both CD68/CD26 (Q2, 3.98%), whereas PSEN1 E280A ChLNs induced an increase in the expression of CD68 (M1), by 35.7% (Q2, Figure 11B). In fact, the expression of CD206 remained almost unchanged in WT iMG + WT PSEN1 ChLNs (Q3: 43.9%, Figure 11A) compared to WT iMG + PSEN1 E280A ChLNs (Q3: 45.5% Figure 11B); therefore, the high percentage of CD68-positive cells was provided by a shift from resting iMG cells (Q4: 51.3%, Figure 11A) to a reactive iMG cell population (Q4: 18.5%, Figure 11B). Not surprisingly, the CD68/CD206 ratio is higher when iMGs are co-cultured with PSEN1 E280A ChLNs (4.5-fold) than with WT ChLNs (Figure 11C). Consistent with these findings, we found that PSEN1 E280A ChLNs induced the release of IL-6 (1.71-fold increase, Figure 11D) and TNF-α (709-fold increase, Figure 11E) by iMG compared to WT ChLNs + iMG. In contrast, IL-10 release by WT iMG was 2.81-fold increased when co-cultured with WT ChLNs compared to PSEN1 E280A ChLNs (Figure 11F).

### 2.17. Presenilin 1 (PSEN1) E280A Cholinergic-like Neurons (ChLNs) Induce a Significant Increase of Pro-Inflammatory M1 Cells (Cluster of Differentiation 68 (CD68)/CD206 Ratio) in PSEN1 E280A and Sporadic AD (SAD) Induced Microglia-like Cells (iMG)

**Figure 12:**
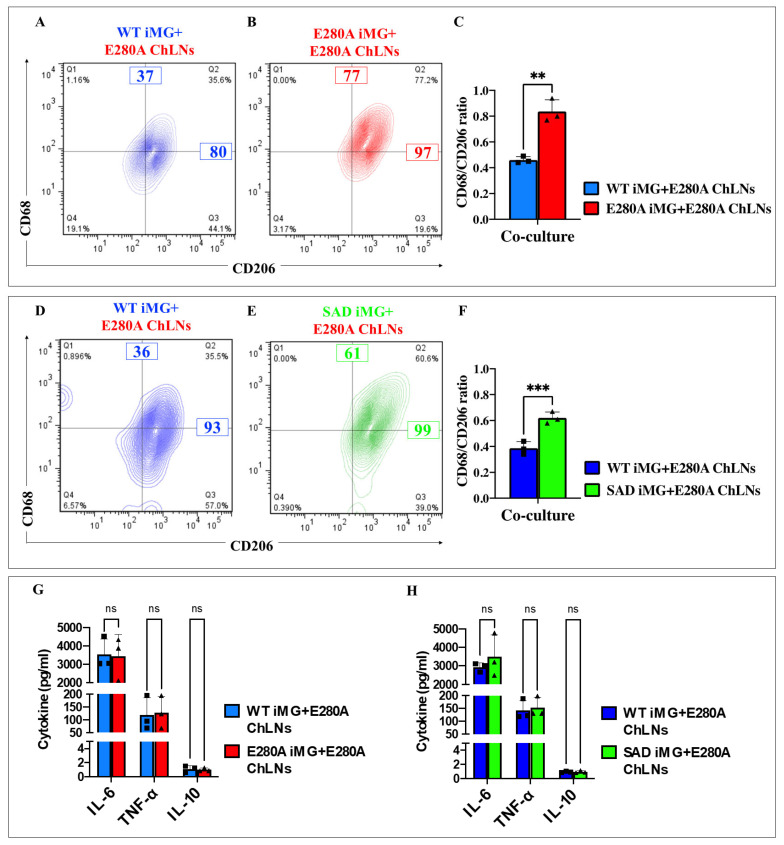
Familial Alzheimer’s disease (FAD, presenilin 1 (PSEN1) E280A;) and sporadic Alzheimer’s disease (SAD) Induced Microglia-like Cells (iMG) show an inflammatory profile and release interleukin-6 (IL-6) and Tumor necrosis factor alpha (TNF-α) but not IL-10 after co-culture with PSEN1 E280A cholinergic-like neurons (ChLNs). iMG cells were induced for 15 days from peripheral blood monocytes obtained from FAD and SAD patients or age- and sex-matched controls. The cells were then co-cultured with PSEN1 E280A ChLNs for 24 h, as described in the Section 4. Representative contour images showing cluster of differentiation 206 (CD206, *x*-axis) and CD68 (*y*-axis) double analysis of age/sex-matched iMG control (blue contour, (**A**) and FAD iMG (red contour, (**B**) co-cultured with PSEN1 E280A ChLNs. (**C**) Quantitative analysis of CD68/CD206 ratio (quadrants Q1 + Q2/quadrants Q2 + Q3). Representative contour images showing CD206 (*x*-axis) and CD68 (*y*-axis) double analysis of age/sex-matched iMG control (blue contour, (**D**) and SAD iMG (green contour, (**E**) co-cultured with PSEN1 E280A ChLNs. (**F**) Quantitative analysis of CD68/CD206 ratio (quadrants Q1 + Q2/quadrants Q2 + Q3). Quantitative analysis of IL-6, TNF-α, and IL-10 release in age/sex-matched iMG control (cyan blue bars) and FAD iMG (red bars) after 24 h (**G**). Quantitative analysis of IL-6, TNF-α, and IL-10 release in age/sex-matched iMG control (cobalt blue bars) and FAD iMG (green bars) after 24 h (**H**). Data are expressed as mean ± SD. ** *p* < 0.01; *** *p* < 0.001; ns = not significant. Figures and bars represent one of three independent experiments (*n* = 3).

We were then interested to establish whether mutant ChLNs in co-culture with PSEN1 E280A and SAD iMG induced pro-inflammatory M1 phenotype in iMG cells. Figure 12 shows that PSEN1 E280A ChLNs induce WT iMG cells (Figure 12A) and PSEN1 E280A iMG cells (Figure 12B) to express the M1 (CD68/CD206) in both WT and mutant iMG by 36% and 77%, respectively. However, PSEN1 E280A ChLNs cells induced a 1.8-fold increase in the expression of M1 (CD68/CD206) marker in PSEN1 E280A iMG compared to WT iMG (Figure 12C). A similar trend was observed when PSEN1 E280A ChLNs were co-cultured with SAD iMG cells (Figure 12D–F).

### 2.18. Presenilin 1 (PSEN1) E280A Cholinergic-like Neurons (ChLNs) Induce a Significant Increase in the Pro-Inflammatory Cytokines Interleukin-6 (IL-6) and Tumor Necrosis Factor Alpha (TNF-α) but Reduce the Anti-Inflammatory IL-10 in Sporadic AD (SAD) and PSEN1 E280A Induced Microglia-like Cells (iMG)

To further characterize the effect of PSEN1 E280A ChLNs on iMG, we examine the secretion of pro-inflammatory cytokine IL-6 and TNF-α, and anti-inflammatory IL-10. As shown in Figure 12, PSEN1 E280A ChLNs induced the secretion of IL-6, TNF-α, and IL-10 to a similar extend in WT iMG, PSEN1 E280A iMG, or SAD iMG (Figure 12G,H).

### 2.19. Presenilin 1 (PSEN1) E280A and Sporadic AD (SAD) Induced Microglia-like Cells (iMG) Induce Abnormal Phosphorylation of Protein TAU and Cleaved Caspase (CC3) in PSEN1 E280A Cholinergic-like Neurons (ChLNs)

**Figure 13:**
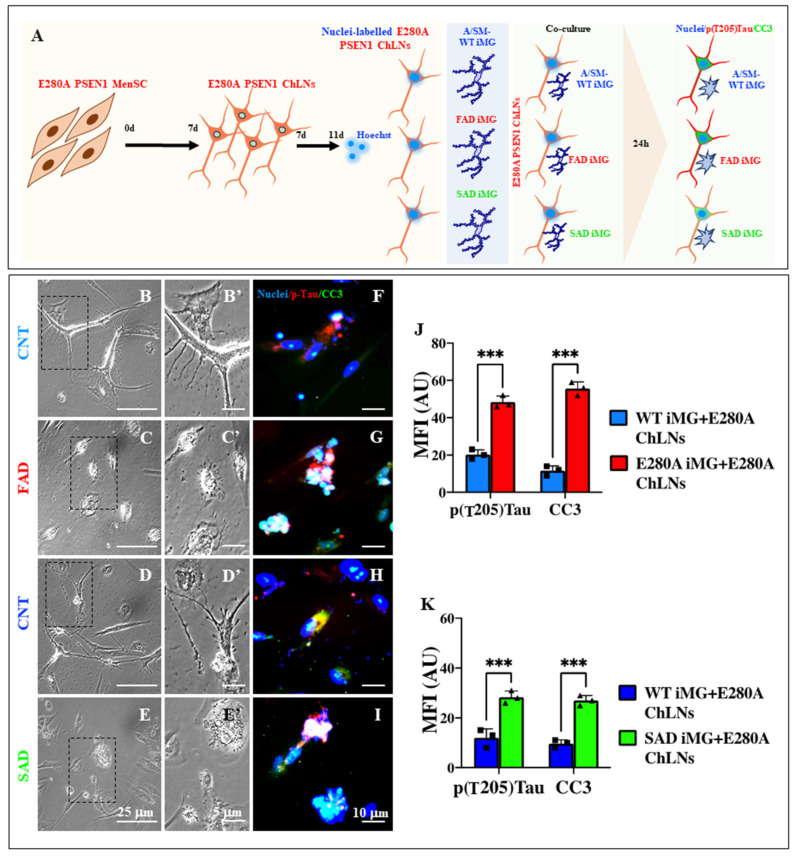
Familial Alzheimer’s disease (FAD, presenilin 1 (PSEN1) E280A) and sporadic Alzheimer’s disease (SAD) Induced Microglia-like Cells (iMG) increase cleavage (activation) of caspase-3 (CC3) and tau (Threonine 205, Thr205) phosphorylation after co-culture with PSEN1 E280A ChLNs. (**A**) iMG cells were induced for 15 days from peripheral blood monocytes obtained from FAD and SAD patients or age- and sex-matched (A/SM) controls. The cells were then co-cultured with PSEN1 E280A ChLNs (Hoechst-positive cells) for 24 h, as described in the Section 4. (**B**) Representative light images and insets showing the morphology of PSEN1 E280A ChLNs after co-culture with age/sex-matched (A/SM) wild type (WT) iMG control; (**C**) PSEN1 E280A ChLNs with PSEN1 E280A age/sex-matched iMG; (**D**) PSEN1 E280A ChLNs with WT iMG control; and (**E**) PSEN1 E280A ChLNs with SAD iMG. Figure (**B’**–**E’**) represent the enlarge area of dashed box in (**B**–**E**). (**F**) Representative fluorescence images showing nuclei (blue), CC3 (green), and p(T205) Tau (red) reactivity in PSEN1 E280A ChLNs after co-culturing with age/sex-matched iMG control; (**G**) PSEN1 E280A ChLNs with PSEN1 E280A age/sex-matched iMG; (**H**) PSEN1 E280A ChLNs with WT iMG control; (**I**) PSEN1 E280A ChLNs with age/sex-matched SAD iMG control. (**J**) Quantitative analysis of p(T205) Tau and CC3 in age/sex-matched iMG control (cyan blue bar) and FAD iMG (red bar) after 24 h. (**K**) Quantitative analysis of p(T205) Tau and CC3 in age/sex-matched iMG control (cobalt blue bar) and SAD iMG (green bar) after 24 h. Data are expressed as mean ± SD. *** *p* < 0.001. Figures and bars represent one of three independent experiments (*n* = 3). Fluorescence image magnification, 40×. Light image magnification, 40×.

Finally, we evaluated whether PSEN1 E280A and SAD iMG induce apoptosis in PSEN1 E280A ChLNs in regard to abnormal phosphorylation of protein TAU and cleaved caspase 3 (CC3) (Figure 13A). Accordingly, while WT (CNT) iMG induced low phosphorylation of TAU at residue T205 and CC3 in PSEN1 E280A ChLNs (Figure 13B,F,J), PSEN1 E280A iMG induced a significant increase in pT205 TAU and CC3 by +140% and +367%, respectively, in PSEN1 E280A ChLNs (Figure 13C,G,J). A similar trend was observed when WT and SAD iMG were co-cultured with PSEN1 E280A ChLNs (Figure 13D,H versus Figure 13E,I,K).

## 3. Discussion

**Figure 14:**
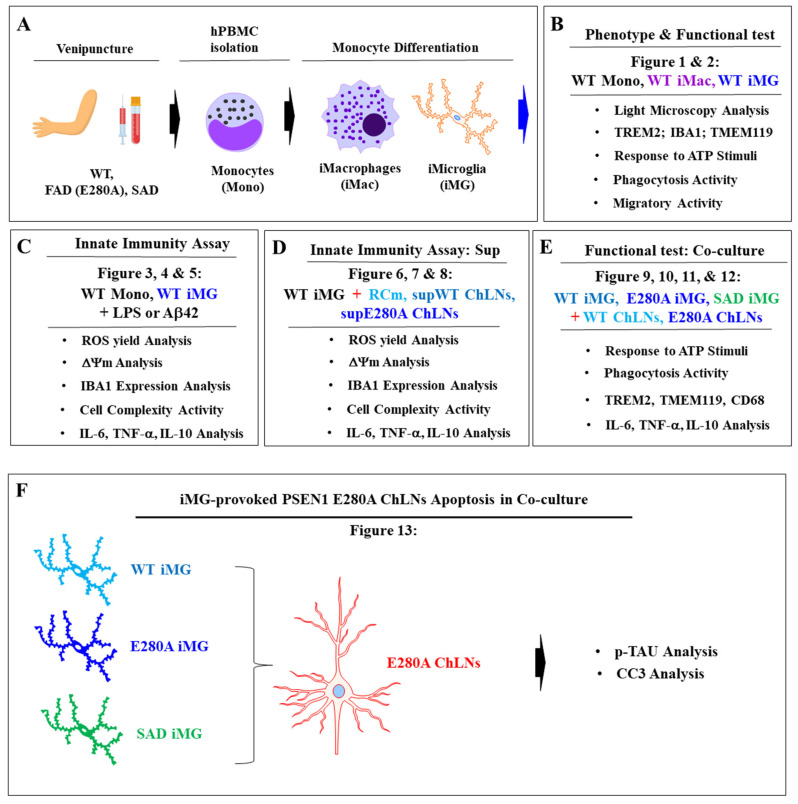
Schematic representation of the Experimental Workflow. (**A**) Isolation and differentiation of human peripheral blood mononuclear cells (hPBMC). Blood samples were obtained by venipuncture from healthy volunteers (wild type, WT), patients with familial Alzheimer’s disease (FAD) bearing the E280A mutation, and patients with sporadic Alzheimer’s disease (SAD). After purification, monocyte (Mono) cells were obtained. Following differentiation of the Mono cells under inducing culture conditions, monocyte-induced macrophages (iMac) and monocyte-induced microglia-like cells (iMG) were obtained. (**B**) WT monocytes, iMacs, and iMGs were characterized by light microscopy analysis according to their phenotype and functional properties (Figure 1 and Figure 2 of this study). These properties included expression of the surface markers triggering receptor expressed on myeloid cells 2 (TREM2), ionized calcium-binding adapter molecule 1 (IBA1), and transmembrane protein 119 (TMEM119); response to ATP stimuli; phagocytic activity; and migratory activity. (**C**,**D**) Wild type (WT) Mono and WT iMG were exposed to lipopolysaccharide (LPS) or amyloid beta 42 (Aβ42) (**C**), and WT iMG were exposed to regular culture medium (RCm) and WT cholinergic-like neuron (ChLN) and presenilin 1 (PSEN1) E280A ChLN supernatant (**D**). The innate immune response was evaluated (shown in Figure 3, Figure 4, Figure 5, Figure 6, Figure 7 and Figure 8) according to reactive oxygen species (ROS) yield, mitochondrial membrane potential (ΔΨm), IBA1 protein expression, cell complexity, and interleukin-6 (IL-6), tumor necrosis factor-alpha (TNF-α), and IL-10 secretion. (**E**) Wild-type (WT) iMG, PSEN1 E280A iMG, and SAD iMG co-cultured with WT ChLNs or PSEN1 E280A ChLNs were evaluated (shown in Figure 9, Figure 10, Figure 11 and Figure 12) according to their response to ATP stimuli, phagocytic activity, and expression of TREM2, TMEM119, and CD68. Secretion of IL-6, TNF-α, and IL-10 was also evaluated. (**F**) PSEN1 E280A ChLNs co-cultured with WT iMG, FAD (PSEN1 E280A) iMG, and SAD iMG were evaluated according to phosphorylated tau (p-tau) and cleaved caspase 3 (CC3) (shown in Figure 13).

In this investigation, we confirm the in vitro monocyte (mono)-induced microglia-like (iMG) cell model using morphology, immunocytochemistry, and functional studies. Consistent with others, iMG cells exhibited the typical morphology of ramified microglia-small soma with extensive radial ramifications [44,45,46], expressed the specific resident microglial marker TMEM119 [61,62,63], and shared myeloid lineage markers IBA1 and TREM2 with iMac. We also verified that iMG cells were functionally active, as (i) they efficiently phagocytosed the red *E. coli* BioParticles™ conjugate (pHrodo™ assay); (ii) iMG efficiently responded to ATP-induced Ca^2+^ influx; and (iii) iMG cells highly migrated in response to a scratch wound stimulus [54]. Furthermore, in response to LPS and bolus addition of neurotoxic Aβ42, iMG cells generated high levels of intracellular ROS [64], increased ∆Ψm, express high cell surface IBA1 marker, and increase cell volume, thereby increasing actin protein expression and cytoskeletal complexity. Importantly, LPS and Aβ42, through activation of transcription NF-κB [65], induced the release of the pro-inflammatory cytokines IL-6 and TNF-α, but the anti-inflammatory IL-10 was not detected in iMG. Taken together, these observations suggest that monocyte-iMG are physiologically similar to human microglia [66]. Therefore, we used mono-iMG as an optimal tool [67] to investigate the pathophysiology of PSEN1 E280A iMG and their interactions with PSEN1 E280A Ch neurons (Figure 14).

Previous studies have shown that PSEN1 E280A ChLNs secrete ~300–620 pg/mL Aβ42 into the culture medium [40,41]. To the best of our knowledge, this study is the first to report that PSEN1 E280A ChLNs supernatant induced important physiological changes in WT iMG cells, such as high production of ROS, increased ΔΨm, expression of high levels of IBA1, and an increase in the extension process (i.e., showing multiple branching) due to increased expression and reorganization of actin protein. Most importantly, PSEN1 E280A ChLNs supernatant induced a change in iMG cellular state from a basal proportion of CD68/CD206 (surveillance state) to a proactive or cell-engaging state favoring CD68 over CD206, known as the cytotoxic M1 phenotype [60]. In addition, PSEN1 E280A ChLNs supernatant also induces WT iMG to release of IL-6 and TNF-α but not IL-10. Taken together, these observations suggest that PSEN1 E280A ChLNs supernatant induces a cellular microglial phenotype in iMG similar to that induced by LPS and Aβ42.

For the first time, we report that both PSEN1 E280A iMG and SAD iMG were almost equally unresponsive to ATP-induced Ca^2+^ influx, and both exhibited defective phagocytic activity. This last alteration was most likely due to reduced TREM2 protein expression. This observation could be explained by the fact that mutated PSEN1 or its inhibition may lead to a reduction in microglial phagocytic activity towards, for example, Aβ [68] or dysfunction of microglial phagolysosomes [69]. Furthermore, when co-cultured with PSEN1 E280A ChLNs, WT iMG, PSEN1 E280A, and SAD iMG cells express a high CD68/CD206 ratio (M1) and secrete high amounts of pro-inflammatory IL-6 and TNF-α but reduced levels of anti-inflammatory IL-10. Taken together, these results suggest that PSEN1 E280A ChLNs are capable of inducing a strong immunological, i.e., microglial M1 phenotype [58], response, most likely triggered by secreted Aβ42 fragments [70].

In agreement with others (e.g., ref. [29]), we showed that WT or PSEN1 E280A microglia overstimulated by noxious stimuli (e.g., Aβ42 fragments, ChLNs-derived supernatant, or ChLNs-secreted Aβ42 [71]), iMG cells undergo a conversion toward an M1 (CD68 high) phenotype, which is responsible for excessive secretion of inflammatory factors, such as IL-6 and TNF-α. Here, we report for the first time that PSEN1 E280A or SAD iMG induced cell death of PSEN1 E280A ChLNs, prompted by the neuronal cells themselves via Aβ42-signaling, most probably through TNF-α mechanism [72]. This instigates a vicious cycle between damaged neurons and M1 phenotypic microglia, resulting in excessive ChLNs death as evidence by pT205 Tau- and CC3-positive cell death-associated markers [41].

Apolipoprotein E (APOE) is a crucial protein involved in fat metabolism in the human body that is associated with Alzheimer’s disease (AD) [73]. APOE is functionally polymorphic and has three major allelic variants: APOE*2 (C112, C158), APOE*3 (C112, R158), and APOE*4 (R112, R158). Our research has shown that PSEN1 E280A iMG (APOE*3/*3) and SAD iMG (APOE*3/*4) are largely unresponsive to ATP stimulation. Moreover, these variants exhibit abnormal phagocytic activity and an aberrant inflammatory response, resulting in significant apoptosis in PSEN1 E280A ChLNs (APOE*3/*4). Since APOE*4 increases proinflammatory signatures in microglia and reduces responses to Aβ pathology [74], we predict that PSEN1 E280A iMG with APOE*3/*4 genotype, as it is the case of SAD, or APOE*4/*4 may be more aggressive phenotypically. However, further investigation is needed to confirm this assumption. Nonetheless, the impact of microglia on the clinical phenotype of patients carrying the PSEN1 E280A mutation required further investigation. This is particularly important given the clinical presentation of PSEN1 E280A patients, involving a median age of onset of 35 years (95% CI 30–36) for asymptomatic pre-mild cognitive impairment (MCI), 38 years (37–40) for symptomatic pre-MCI, 44 years (43–45) for MCI, and 49 years (49–50) for dementia. The median age at death is 59 years (95% CI 58–61) [75]. As clinical deterioration can be detected as measurable cognitive impairment around two decades before the onset of dementia in PSEN1 E280A carriers, our findings could potentially serve as a model for exploring the complex interaction between PSEN1 variants and the APOE genotype in microglia in relation to neuron-secreted Aβ.

Since yolk-sac-derived microglia comprise the permanent pool of brain microglia throughout an individual’s lifetime, there are no known limitations in using blood-derived iMG. In line with this view, our hPBMC-derived iMG offers several advantages as a cellular system model over other biological sources, such as embryonic yolk sac-derived MG and induced pluripotent stem cell-derived MG [62,63]. Firstly, obtaining hPBMCs involves a minimally invasive procedure: venipuncture. Importantly, this procedure is age-independent for both volunteers and FAD patients. Secondly, hPBMCs raise minimal bioethical concerns according to ethical review boards. Thirdly, our data demonstrated that the hPBMC-derived iMG model mimicked many features of the brain’s resident microglia, highlighting its utility in the study of microglial function. In agreement with this assumption, comprehensive transcriptome analysis and multidimensional scaling analyses of RNA-seq data have demonstrated that hPBMC-derived iMG are similar to brain primary microglia [45,76]. Moreover, a comparison of neuronal, microglial, and macroglial (astrocyte and oligodendrocyte) markers reveals that profiles in brain organoids are similar to autopsied human cortical and cerebellar profiles, providing the first demonstration that human-specific protein processing is largely conserved in unguided brain organoids [77]. Finally, the hPBMC-derived iMG methodology and monocyte differentiation protocols are straightforward, cost-effective, and save laboratory time. Accordingly, hPBMC-derived iMG can be obtained within 15 days. Therefore, hPBMC-derived iMG are an excellent platform to test potential drug targets [78] and drug candidates [79] in AD/FAD.

## 4. Materials and Methods

### 4.1. Generation of Induced Microglia-like Cells (iMG Cells) from Human Monocytes

Induced MG cells were obtained according to [45], with minor modifications. Briefly, human peripheral monocyte cells (PBMCs) were isolated from age-matched healthy fresh blood individuals (2–3 × 10^6^ cells/cm^2^; tissue bank code (TBC) # PBMC0001, PBMC0002), from familial Alzheimer’s disease harboring PSEN1 E280A mutation (FAD; PSEN1 E280A, TBC # PBMC1117), and sporadic AD (SAD, TBC # PBMC21513) (Table 1) by density gradient centrifugation with Histopaque-1077 (SIGMA, cat# 10771; Merck KGaA, Darmstadt, Germany). hPBMCs were cultured in serum-free media (RPMI-1640-Glutamax, cat# 61870036, Gibco, New York, NY, USA) alone or serum-free media supplemented with 0.1 μg/mL IL-34 (recombinant human, cat# ab288525, ABCAM, Cambridge, MA, USA), 0.01 μg/mL GM-CSF (recombinant human GM-CSF protein (Active), ABCAM, cat# ab259376, Cambridge, MA, USA), and 1% P/S (MP Biomedicals, cat# 091674049, Santa Ana, CA, USA). Induced macrophages (iMac, TBC # PBMC0001) were also generated from monocytes by induction with RPMI-1640 Glutamax media containing 10% FBS (SIGMA, cat#12103C, Merck KGaA, Darmstadt, Germany), 0.01 μg/mL GM-CSF, and 1% P/S for 7 days. During the induction, we replaced fresh media every 3–4 days.

### 4.2. Evaluation of Cell Morphology

After induction, cellular morphology was monitored with phase-contrast microscopy (Zeiss Axiovert A1 microscope, Zeiss Wöhlk, Gmbh, Schoenkirchen, Germany). Images were captured with a Zeiss Axiocam CM1 camera (Zeiss Wöhlk, Gmbh, Schoenkirchen, Germany) through ZEN 3.9 Imaging Software (https://www.zeiss.com/microscopy/en/products/software/zeiss-zen.html, accessed on 20 January 2025).

### 4.3. Flow Cytometry Analysis of Microglia Markers

To determine general microglia markers [60], we initially used flow cytometry analysis. Briefly, 1 × 10^5^ monocyte or iMG cells were detached and fixed with cold ethanol (80%) overnight and washed thrice with PBS. Cells were then blocked with 5% BSA (Cat. A8806, SIGMA, Cat. 10771; Merck KGaA, Darmstadt, Germany)/PBS for 30 min, followed by incubation with primary conjugated anti-TMEM119-488 (Rabbit, cat. Ab225497, ABCAM, Cambridge, MA, USA), TREM2 (Rabbit, cat. 702886, Invitrogen, Carlsbad, CA, USA) and IBA1 (Ionized calcium binding adapter molecule 1, IBA1; Mouse, cat. SAB2702364, Sigma-Aldrich, St. Louis, MO, USA) antibodies (1:100; 4 °C overnight). Depending on the species of primary antibodies, 1:500 Alexa Fluor 488- or Alexa Fluor 594-conjugated antibodies were used for completing the staining (Donkey, Cat. A21206 and A21203, Invitrogen, Carlsbad, CA, USA). Finally, cells were washed and re-suspended in PBS for analysis on a BD LSRFortessa II flow cytometer (BD Biosciences, Franklin Lakes, NJ, USA). In total, 10,000 events were acquired, and the acquisition analysis was performed using FlowJo v10.10 Data Analysis Software (https://www.flowjo.com, accessed on 20 January 2025).

### 4.4. Immunofluorescence Analysis of Microglia Markers

For fluorescence microscopy analysis, monocyte or iMG cell culture (24-well plates) was fixed with 4% formaldehyde overnight and washed thrice with PBS. Cells were then permeabilized and blocked with 0.2% Triton X-100 plus 5% BSA/PBS for 60 min, followed by incubation with primary conjugated anti-TMEM119-488 nm; and with primary unconjugated TREM2, and IBA1 antibodies (1:100; 4 °C overnight). Depending on the species of primary antibodies, 1:500 Alexa Fluor 488- or Alexa Fluor 594-conjugated antibodies were used for completing the staining. The nuclei were stained with (0.5 μM) Hoechst 33342 (Life Technologies, Carlsbad, CA, USA), and images were acquired on a fluorescent microscope (Zeiss Axiovert A1 microscope, Zeiss Wöhlk, Gmbh, Schoenkirchen, Germany). Images were captured with a Zeiss AxioCam CM1 camera (Zeiss Wöhlk, Gmbh, Schoenkirchen, Germany).

### 4.5. Assessment of Microglial Function by Calcium Activity

Calcium activity was performed as described previously with minor modifications [80]. For the measurement, the fluorescent dye Fluo-3 (Fluo-3 AM; Thermo Fisher Scientific, cat# F1242, Waltham, MA, USA) was employed. The dye was dissolved in DMSO (1 mM, SIGMA, cat#472301, Merck KGaA, Darmstadt, Germany) as a stock solution. Before the experiments, the stock solution was diluted in neuronal buffer solution (NBS buffer: 137 mM NaCl, 5 mM KCl, 2.5 mM CaCl_2_, 1 mM MgCl_2_, pH 7.3). The working concentration of the dye was 2 μM. Monocyte or iMG cells were seeded approximately 80% confluent and were incubated for 30 min at 37 °C with the dye containing NBS and then washed five times. The intracellular Ca^2+^ transients were evoked by ATP (adenosine triphosphate; Sigma-Aldrich, Cat. A7699, St. Louis, MO, USA; 1 mM final concentration). The measurements were carried out using the 20× objective of a fluorescent microscope (Zeiss Axiovert A1 microscope, Zeiss Wöhlk, Gmbh, Schoenkirchen, Germany) equipped with a Zeiss Axiocam CM1 camera (Zeiss Wöhlk, Gmbh, Schoenkirchen, Germany). Several regions of interest (ROIs) were defined in the visual field of the camera. One of the ROIs was cell-free, and the fluorescence intensity measured here was considered as background fluorescence (F_bg_). The time dependence of the fluorescence emission was acquired, and the fluorescence intensities (hence the Ca^2+^ levels) were represented by pseudocolors. To calculate the changes of the average Ca^2+^-related fluorescence intensities, first the F_bg_ value was determined from the cell-free ROI, and then the resting fluorescence intensities (F_rest_) of the cell-containing ROIs were obtained as the average of the points recorded during a period of 10 s prior to adding ATP. The peaks of the fluorescence transients were found by calculating the average of six consecutive points and identifying those points that gave the highest average value (F_max_). The amplitudes of the Ca^2+^-related fluorescence transients were expressed relative to the resting fluorescence (ΔF/F) and were calculated by the following formula: ΔF/F = (F_max_ − F_rest_)/(F_rest_ − F_bg_). For the calculation of the fluorescence intensities, ImageJ program was used (https://imagej.net/ij/, accessed on 25 January 2025). The term fluorescence intensity was used as an indirect indicator of intracellular Ca^2+^ concentration.

### 4.6. Scratch Wound Migration Assay

A scratch wound migration assay was performed as described previously [81]. Briefly, monocyte or iMG cells were seeded approximately 80% confluent. Then, the monolayer was scratched with a sterile 200 μL pipette tip, and the cells were incubated for a further 24 h to allow time for migration into the cell-free area. We counted all microglia in the scratch region and calculated the mean from 6 to 9 separate cultures. Wound healing analysis was performed by image measurement using ImageJ plugging according to previous reports [82].

### 4.7. Live Flow Cytometry and Imaging Phagocytic Assay with pHrodo^TM^

Live imaging phagocytosis was performed according to Sinha and coworkers [53]. Monocyte or iMG cells were plated in 24-well plates and cultured until 70–80% confluency. The cell media was removed from each well; then wells were washed twice with PBS, and 500 μL of medium containing 1 μL of pHrodo™ (Red *E. coli* BioParticles™ Conjugate for Phagocytosis, cat. #P35361, Thermo Fisher Scientific, Waltham, MA, USA) was added into each well. The plates were kept in the incubator with 5% CO_2_ at 37 °C for 6 h; then the media was removed, and the wells were washed thrice with PBS to remove any unbound pHrodo debris. For flow cytometry, cells were detached using trypsin and re-suspended in PBS for analysis on a BD LSRFortessa II flow cytometer (BD Biosciences, Franklin Lakes, NJ, USA). In total, 10,000 events were acquired, and the acquisition analysis was performed using FlowJo v10.10 Data Analysis Software (https://www.flowjo.com, accessed on 25 January 2025). For fluorescence microscopy analysis, 200 μL of fresh phagocytosis assay medium was added into each well, and then imaging was conducted using a fluorescent microscopy (Zeiss Axiovert A1 microscope, Zeiss Wöhlk, Gmbh, Schoenkirchen, Germany) equipped with a Zeiss Axiocam CM1 camera (Zeiss Wöhlk, Gmbh, Schoenkirchen, Germany).

### 4.8. Human Menstrual Stromal Cells (MenSCs)

The menstrual blood (MenB) samples were collected from one healthy female and one female carrier of the mutation PSEN1 E280A aged 30 years (WT MenSC (CNT) bank tissue code TBC# MSC-MB0001) and 25 years (MenSC E280A, TBC# MSC-MB0002), according to [83] (Table 1). Donors signed an informed consent accepted by the Ethics Committee of the Sede de Investigación Universitaria-SIU-, Act #2020-10854, University of Antioquia, Medellín, Colombia.

### 4.9. Cholinergic-like Neuron (ChLN) Differentiation

ChLN differentiation was performed according to refs. [41,59]. Briefly, WT and mutant MenSCs were seeded at 1–1.5 × 10^4^ cells/cm^2^ in laminin-treated culture plates for 24 h in regular culture medium. Then, the medium was removed, and cells were incubated in cholinergic differentiation medium (Cholinergic-N-Run medium, hereafter Ch-N-Rm) at 37 °C for 7 days, and then the medium was replaced by minimal culture medium (mCM) for 4 days. Then, supernatants were collected, filtrated (0.22 μm Polyethersulfone (PES) membrane, Corning^®^, W Camelback Rd, Glendale, AZ, USA), and stored at −80 °C until use.

### 4.10. Evaluation of Intracellular Hydrogen Peroxide (H_2_O_2_) and Mitochondrial Membrane Potential (ΔΨm) by Fluorescent Microscopy

Monocyte or iMG cells were plated in 24-well plates and cultured until 70–80% confluency. Then, they were left untreated or treated with 100 μg/mL LPS (Sigma, Lipopolysaccharides from *Escherichia coli* O26:B6, St. Louis, MO, USA), 10 μg/mL Aβ42 peptide (Sigma, Cat. A9810, St. Louis, MO, USA), WT PSEN1 ChLNs supernatant, or PSEN1 E280A ChLNs supernatant for 24 h. Then after, cells were incubated with the DCFH_2_-DA (1 μM, DCFH_2_-DA; Invitrogen, Carlsbad, CA, USA) and active mitochondria-accumulating dye deep red MitoTracker^®^ compound (20 nM, final concentration, Thermo Fisher Scientific, cat# M46753, Waltham, MA, USA) for 30 min at 37 °C in the dark. Cells were then washed, and DCF/Mitotracker fluorescence intensity was determined by analyzing fluorescent microscopy images. The nuclei were stained with 0.5 µM Hoechst 33342 staining compound (Life Technologies, Carlsbad, CA, USA).

### 4.11. Evaluation of Intracellular Hydrogen Peroxide (H_2_O_2_) and Mitochondrial Membrane Potential (ΔΨm) by Flow Cytometry

Monocyte or iMG cells were plated in 6-well plates and cultured at 70–80% confluency. Then, they were left untreated or treated with LPS (100 μg/mL), Aβ42 peptide (10 μg/mL), WT PSEN1 ChLNs supernatant, or PSEN1 E280A ChLNs supernatant for 24 h. Then, cells were incubated with DCFH2-DA (1 μM, Sigma, cat#35845, St. Louis, MO, USA) and MitoTracker^®^ (20 nM) reagents for 30 min at 37 °C in the dark. Cells were then washed, and DCF/MitoTracker fluorescence was determined using a BD LSRFortessa II flow cytometer (BD Biosciences, Franklin Lakes, NJ, USA). A total of 10,000 events were acquired, and quantitative data and figures were obtained using FlowJo 7.6.2 Data Analysis Software.

### 4.12. Microglia Process Extension Analysis

Process extension analysis was performed according to ref. [84]. Briefly, Mono or iMG cells were plated in 24-well plates and cultured until 70–80% confluency. Then, they were left untreated or treated with LPS (100 μg/mL) or Aβ42 peptide (10 μg/mL), PSEN1 WT ChLNs supernatant/co-culture, or PSEN1 E280A ChLNs supernatant/co-culture for 24 h. Then, IBA1 staining was performed, and cells were imaged. The binary images were then skeletonized using the Skeletonize 2D/3D Plugin for ImageJ (https://imagej.net/plugins/skeletonize3d, accessed on 25 January 2025). Sparingly, manual segmentation was used to separate a single skeleton that was part of two cells touching each other. The Analyze Skeleton Plugin (https://imagej.net/plugins/analyze-skeleton/, accessed on 25 January 2025) was then applied to the skeletonized images to obtain parameters related to process length and number of branches for each cell in the imaging field. The data was summarized as average process length for a specific imaging field.

### 4.13. Cytokine Release Measurement

Monocyte or iMG cells were plated in 6-well plates and cultured until 70–80% confluency. Then, cells were left untreated or treated with LPS (100 μ/mL), Aβ42 peptide (10 μ/mL), WT PSEN1 ChLNs supernatant or PSEN1 E280A ChLNs supernatant for either 1 h or 24 h. The IL-6, IL-10, and TNF-α secretion levels were measured in cell-free supernatants using Cytometric Bead Array (CBA) of human inflammatory cytokines (BD Biosciences, cat# 551811; Franklin Lakes, NJ, USA) according to the manufacturer’s protocol. Data was acquired using a CytoFLEX Flow Cytometer (Beckman Coulter Life Sciences, Indianapolis, IN, USA).

### 4.14. Co-Culture of iMG and Cholinergic-like Neuron (ChLNs)

WT and/or mutant MenSCs were seeded at 1–1.5 × 10^4^ cells/cm^2^ in laminin-treated 24-well culture plates for 24 h in regular culture medium. Then, the medium was removed, and cells were incubated in cholinergic differentiation medium (Cholinergic-N-Run medium, hereafter Ch-N-Rm) at 37 °C for 7 days. Then, the medium was replaced with minimal culture medium (DMEM low glucose plus 1%FBS) for 4 days. Further, iMG were seeded at 70–80% confluency and incubated for 24 h. For flow cytometry analysis, the ChLNs were stained using goat anti-ChAT antibody (Sigma, Cat. cat# AB144P, St. Louis, MO, USA), and iMG were labeled using TMEM119 antibody (see above). For fluorescence microscopy analysis, the cells were stained using mouse anti-actin antibody (Invitrogen, cat# MA5-11869, Carlsbad, CA, USA), and iMG were labeled using TMEM119 antibody (see above).

### 4.15. Flow Cytometry Analysis of Microglia Inflammatory Phenotype

To determine microglia inflammatory phenotype, we used flow cytometry and fluorescence microscopy analysis. Briefly, after supernatant or co-culture incubation, 1 × 10^5^ iMG cells were detached and fixed with cold ethanol (80%) overnight and washed thrice with PBS. Cells were then blocked with 5% BSA/PBS for 30 min, followed by incubation with primary anti-CD206 (Mouse, Santa Cruz, cat# SC-376232, Dallas, TX, USA) and CD68 (rabbit, ABCAM, cat# Ab277938, Cambridge, MA, USA) antibodies (1:100; 4 °C overnight). Depending on the species of primary antibodies, 1:500 Alexa Fluor 488- or Alexa Fluor 594-conjugated antibodies were used to complete the staining. Finally, cells were washed and re-suspended in PBS for analysis on a BD LSRFortessa II flow cytometer (BD Biosciences, Franklin Lakes, NJ, USA). A total of 10,000 events were acquired, and the acquisition analysis was performed using FlowJo v10.10 Data Analysis Software (https://www.flowjo.com, accessed on 5 February 2025).

### 4.16. Immunofluorescence Analysis of Tau and Cleaved Caspase-3 (CC3)

Mutant MenSCs were seeded at 1–1.5 × 10^4^ cells/cm^2^ in laminin-treated 24-well culture plates in regular culture medium for 24 h. The medium was then removed, and the cells were incubated in cholinergic differentiation medium (cholinergic N-Run medium) at 37 °C for 7 days. The medium was then replaced with minimal culture medium (DMEM low glucose plus 1% FBS) for 4 days. The ChLNs nuclei were then labeled with Hoechst 33342). After several washes, nuclei unstained-iMG and nuclei labelled-ChLNs were co-cultured for 24 h as described above, and then cells were fixed. For the analysis of neuronal Alzheimer’s disease and cell death-related markers, cells were incubated overnight with primary antibody anti-phospho (T205) Tau (Rabbit, ABCAM, cat# Ab307376, Cambridge, MA, USA) and 1X CellEvent Caspase3/7 Detection Reagent (Invitrogen, cat# C10423, Carlsbad, CA, USA). Fluorescence microscopy images were captured using a Zeiss Axio Vert.A1 fluorescence microscope equipped with a Zeiss AxioCam Cm1 (Zeiss Wöhlk-Contact-Linsfluoreen, Gmbh, Schoenkirchen, Germany).

### 4.17. Apolipoprotein E Genotype

The APOE genotype assessment was performed by LIME (Laboratorio Integrado de Medicina Especializada) laboratory at University of Antioquia.

### 4.18. Data Analysis

In this experimental design, peripheral blood mononuclear cells were isolated and cultured, and the cell suspension was pipetted at a standardized cellular density of 2–3 × 10^6^ cells per cm^2^ into different wells of a 24- or 6-well plate. Cells (i.e., the biological and observational units) [85] were randomized to wells by simple randomization (sampling without replacement method), and then wells (i.e., the experimental units) were randomized to treatments using a similar method. Experiments were conducted in triplicate. The data from individual replicate wells were averaged to yield a value of *n* = 1 for the experiment, and this was repeated on three occasions blind to the experimenter and/or flow cytometer analyst for a final value of *n* = 3 [85]. Based on the assumptions that the experimental unit (i.e., the well) data comply with independence of observations, the dependent variable is normally distributed in each treatment group (Shapiro–Wilk test), and there is homogeneity of variances (Levene’s test); the statistical significance was determined by one-way or two-way analysis of variance (ANOVA) followed by Tukey’s post hoc comparison to compare the differences between the multiple experimental groups. Moreover, Student’s *t*-test was used to compare the differences in a particular group (phagocytic index). All statistical analyses were calculated with GraphPad Prism 5.0 software (https://www.graphpad.com/, accessed on 18 January 2025). Differences between groups were only deemed significant when a *p*-value of 0.05 (*), 0.01 (**), or 0.001 (***) was present. All data were illustrated as the mean ± SD or ± SEM (only for the results of the functional analysis of ATP response).

## 5. Conclusions

Using a culture medium containing IL-34 (100 ng/mL) and GM-CSF (10 ng/mL) for 15 days, we obtained mature induced microglia-like cells from hPBMCs. The iMG cells displayed the typical functional phenotype of microglial cells. Indeed, upon exposure to LPS, Aβ42, or PSEN 1 ChLNs-derived culture supernatant, iMG were able to generate ROS, increase ΔΨm and IBA1 reactivity markers, increase cell body size resembling an amoeboid-like phenotype, show a disproportionate expression of proinflammatory CD68 over CD206 ratio typical of M1 microglial cells, and secrete high levels of proinflammatory IL-6 and TNF-α. Interestingly, iMG derived from PBMCs of a FAD patient carrying PSEN1 E280A mutation and a sporadic case of AD were physiologically altered to a similar extent. Indeed, both iMG cells were unresponsive to ATP-induced transient Ca^2+^ influx and were deficient in phagocytic activity compared to WT iMG cells. In co-culture with PSEN1 E280A ChLNs, PSEN1 E280A and SAD iMG displayed typical features of active microglia, such as high expression of the CD68 marker (M1) and high secretion of IL-6 and TNF-α, but reduced secretion of IL-10. In turn, iMG induced apoptosis in PSEN1 E280A ChLNs (i.e., pT205 Tau- and CC3-positive cells). Understanding this interaction is critical for the development of targeted therapies that can modulate microglial activity and potentially slow disease progression in FAD. Given that this investigation is based on a single blood sample from two age-matched healthy (WT) individuals, one familial (PSEN1 E280A) and one SAD case, our results should be considered exploratory. Larger sample sizes are needed. Nonetheless, future research will determine whether increasing PSEN1 E280A microglial phagocytic activity [86], attenuating TNF-α signaling [87], or directly targeting microglia (e.g., [88,89]) could serve to delay, halt, or reduce neuronal damage in FAD.

## Figures and Tables

**Table 1:** List of samples, cell type, differentiated cell type, age, sex and APOE genotype of the biological samples used in this study.

Sample	TBC#	Cell Type	Differentiated Cell Type	Age	Sex	APOE*/*Genotype
WT (CNT)	PBMC0001	PBMC	WT iMG	39	Male	3/3
FAD E280	PBMC1117	PBMC	E280A iMG	45	Male	3/3
WT (CNT)	PBMC0002	PBMC	WT iMG	82	Male	3/3
SAD	PBMC21513	PBMC	SAD iMG	75	Male	3/4
WT MenSC	MSC-MB0001	MenSC	WT ChLNs	23	Female	3/3
MenSC E280A	MSC-MB0002	MenSC	E280AChLNs	25	Female	3/4

Abbreviations. CNT, control; FAD, familial Alzheimer’s disease; SAD, sporadic Alzheimer’s disease; MenSC, menstrual stromal cells; E280A, variant; TBC#, tissue bank code#; WT, wild-type; iMG, induced microglia-like cells; APOE*, APOE allele.

## Data Availability

All datasets generated for this study are included in the manuscript.
